# Spatiotemporal Adaptations‐Driven Dynamic Thra Activation Simulates a Skin Wound Healing Response

**DOI:** 10.1002/advs.202506651

**Published:** 2025-06-25

**Authors:** Zeming Li, Jiajun Tan, Chuqing Zhou, Siyi Zhou, Yuanli Ye, Xinzhu Li, Xinyu Shen, Tian Xie, Mengyue Wang, Jingwei Jiang, Yiping Zhao, Xiao Xiang, Yong Zhou, Jun Wu, Cheng‐Ming Chuong, Mingxing Lei

**Affiliations:** ^1^ Key Laboratory of Biorheological Science and Technology of Ministry of Education & 111 Project Laboratory of Biomechanics and Tissue Repair College of Bioengineering Chongqing University Chongqing 400044 China; ^2^ Chongqing Weisiteng Biotech Transnational Research Institute Chongqing 400039 China; ^3^ Department of Burn and Plastic Surgery Shenzhen Institute of Translational Medicine Shenzhen Second People's Hospital The First Affiliated Hospital of Shenzhen University Shenzhen 518035 China; ^4^ Department of Pathology Keck School of Medicine University of Southern California Los Angeles CA 90033 USA

**Keywords:** glutathione metabolism, SAA3, skin organoids, thyroid hormone, wound healing

## Abstract

The evolutionary adaptation of skin repair drives sequential regenerative phases: epidermal proliferation rapidly restores barrier function, followed by dermal reconstruction through extracellular matrix remodeling to establish structural support, yet the molecular coordination of this spatiotemporal program remains unclear. While the endocrine system is crucial in modulating wound repair, the critical hormone receptors orchestrating tissue‐layer‐specific responses are unidentified. Here, bulk and single‐cell RNA sequencing, spatial transcriptomics, and in vivo/in vitro analyses in mouse models of hyperthyroidism and hypothyroidism, as well as wound and skin organoid models, are employed to identify the thyroid hormone receptor Thra as a key regulator of phase‐coupled regeneration through two distinct yet coordinated mechanisms. In the initial phase, epidermal Thra activates glutathione metabolism via Gamma‐Glutamylcyclotransferase (GGCT), driving keratin filament assembly to accelerate reepithelialization. In the subsequent phase, dermal Thra mediates the Serum Amyloid A3 (SAA3)‐Fibronectin 1 (FN1) interaction, establishing angiogenic niches essential for matrix maturation. Using the self‐assembled epidermis‐dermis dynamic skin organoid model, Thra's role in simulating the wound healing process is further confirmed. This study highlights the essential role of spatiotemporal adaptability in wound repair using Thra as a paradigm and provides insights for developing clinical strategies to enhance skin wound healing.

## Graphical abstract

This study uncovers the thyroid hormone receptor Thra as a pivotal regulator that spatiotemporally orchestrates dual‐phase skin repair. Epidermal Thra accelerates barrier restoration via glutathione‐driven keratin assembly, while dermal Thra establishes angiogenic niches through SAA3‐FN1‐mediated matrix remodeling. Integrated transcriptomic, spatial, and organoid analyses reveal Thra's phase‐coupled adaptability, offering insights for enhancing regenerative therapies by coordinating layered tissue regeneration dynamics.

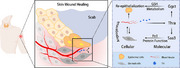

## 1 Introduction

Spatiotemporal adaptations are evolutionarily conserved strategies that coordinate dynamic biological processes across distinct tissue layers and developmental phases. During liver regeneration, hepatocytes and stromal cells exhibit spatiotemporally distinct behaviors: rapid parenchymal proliferation precedes ECM‐driven architectural restoration.^[^ ^1^, ^2^ ^]^ This highlights that layered tissues often compartmentalize regeneration into sequential programs, first addressing immediate functional demands such as barrier integrity, followed by long‐term structural reinforcement. Importantly, such spatiotemporally coordinated regeneration is exemplified in skin wound healing—the epidermis prioritizes rapid reepithelialization to prevent infection, while the dermis requires delayed yet precise ECM remodeling to restore mechanical strength.^[^ ^3^, ^4^ ^]^ However, the molecular machinery coordinating this spatiotemporal program remains elusive.

Building on this concept of spatiotemporally compartmentalized regeneration, skin wound healing provides an exemplary model where layered tissues coordinate temporally distinct repair phases. The process begins with inflammation where immune cells clear pathogens and debris, followed by the proliferative phase in which fibroblasts and keratinocytes regenerate tissue, produce collagen, and restore the epidermal barrier.^[^ ^3^, ^5^, ^6^ ^]^ Throughout these spatiotemporally segregated stages, the endocrine system has emerged as a crucial spatiotemporal coordinator of skin repair mechanisms, modulating phase‐specific inflammatory responses and proliferative programs via hormonal signaling.^[^ ^7^, ^8^, ^9^ ^]^ Among these, thyroid hormone (TH), a key evolutionary conserved endocrine signal,^[^ ^10^ ^]^ has garnered significant attention for its pan‐tissue regulatory capacity in physiological remodeling. Notably, the thyroid hormone pathway has been implicated in orchestrating spatiotemporal tissue remodeling across diverse biological contexts, including amphibian metamorphosis,^[^ ^11^, ^12^ ^]^ skeletal maturation,^[^ ^13^, ^14^ ^]^ and cardiac development.^[^ ^15^ ^]^ For instance, TH has been shown to drive extracellular matrix (ECM) reorganization and matrix metalloproteinase (MMP) activity during Xenopus gut metamorphosis.^[^ ^16^ ^]^ Additionally, TH has been demonstrated to stimulate epithelial cell proliferation and migration in wound healing, processes that are critical for restoring tissue integrity.^[^ ^17^, ^18^, ^19^ ^]^ A recent study revealed that radiation‐induced hypothyroidism is associated with poor surgical outcomes in salvage laryngectomy, linking low thyroid hormone levels to impaired wound healing.^[^ ^20^ ^]^ Collectively, these findings suggest that thyroid hormone may play a pivotal role in skin wound healing by modulating ECM reorganization and promoting epithelial cell functions.

**Figure 1:**
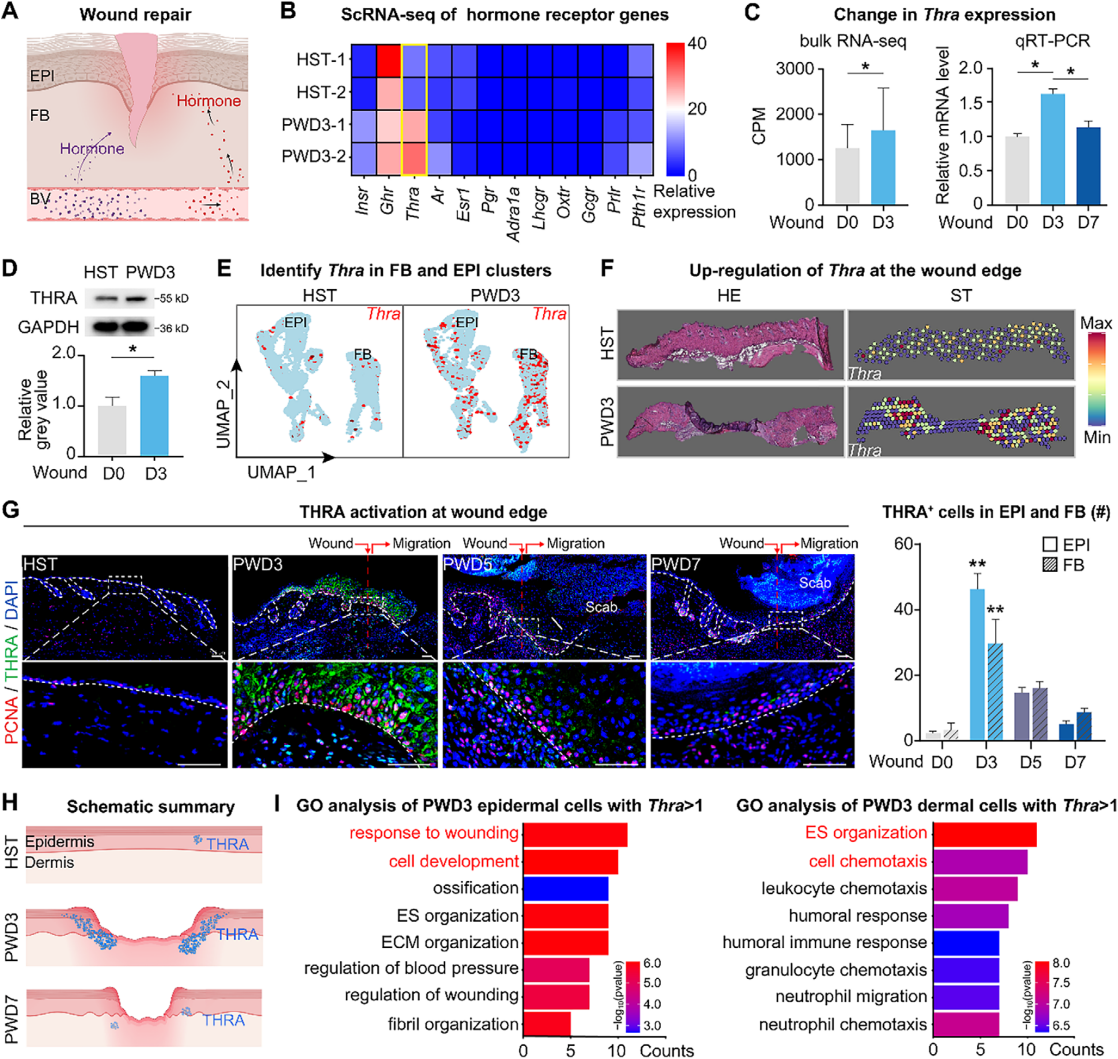
Spatiotemporal expression of THRA upregulated on post‐wounding day 3. A) Illustration of the influence of different hormones on the wound repair process. B) Heat Plot of the expression of different hormone receptors in HST and PWD3 skin. C) Bulk RNA‐seq analysis of Thra expression levels on day 0 and 3 post‐wounding; qRT‐PCR analysis of Thra expression levels on day 0, 3, and 7 post‐wounding. (N = 3, ^*^p < 0.05). D) FeaturePlots of the expression of Thra in HST and PWD3 skin within FB and EPI clusters. E) Western blotting analysis of THRA protein levels on D0 and PWD3. (N = 3, ^*^p < 0.05). F) Spatial transcriptomics data of the expression of Thra in HST and PWD3 skin. G) Immunofluorescence images of THRA expression on PWD3, with statistical analysis of THRA^+^ cells in the epidermis and dermis. (Scale bars, 100 µm; N = 5, ^*^ ^*^p < 0.01). H) Schematic summary of the expression of Thra post‐wounding. I) GO analysis of pathways enriched in Thra‐positive cells in the epidermis and dermis post‐wounding.

Given the established role of thyroid hormone in tissue remodeling and wound healing, elucidating its specific mechanisms in skin wound healing is of significant interest. Thyroid hormone, a key endocrine regulator, is primarily secreted by the thyroid gland (**Figure** **1**A). Once released into the bloodstream, it travels to distant target cells and tissues, binding to nuclear receptors, TRα and TRβ, which function as ligand‐inducible transcription factors to regulate the expression of target genes.^[^ ^21^ ^]^ In the context of skin wounds, vasodilation of blood vessels increases the delivery of biomolecules to the wound site, thereby enhancing the interaction of thyroid hormone with local cells and tissues. Thyroid hormone has been implicated in various cellular functions essential for wound healing, including metabolism, growth, differentiation, and immune response. However, the precise spatiotemporal mechanisms by which thyroid hormone influences these processes in skin wound healing remain unclear. This knowledge gap is particularly critical given the potential therapeutic applications of modulating thyroid hormone signaling in coordinating layered tissue regeneration.

To delineate the spatiotemporal dynamics of thyroid hormone (TH)‐mediated skin repair, our work investigated the TH‐induced skin‐layer‐specific repair mechanisms during skin wound healing by employing mouse models of hyperthyroidism and hypothyroidism, mouse wound models, and skin organoid models. Utilizing spatial transcriptomics, we revealed that the thyroid hormone receptor gene, Thra, exhibited a layered spatiotemporal expression pattern–temporally and spatially upregulated in both the dermal and epidermal layers following skin injury. In the epidermis, activation of Thra by TH influenced the expression of γ‐glutamylcyclotransferase (Ggct), thereby regulating glutathione metabolism in epidermal cells. This metabolic regulation subsequently affected the “cornified envelope” and “keratin filament” structures of epidermal cells, promoting epithelial regeneration. In the dermis, TH‐activated Thra upregulated serum amyloid A3 (Saa3) expression and interacted with fibronectin 1 (Fn1), thereby regulating vascular development and regeneration. These findings provide novel insights into the hormone‐induced regulatory network driving spatiotemporally repair in skin wound healing and highlight the critical role of Thra in coordinating phase‐specific epidermal and dermal recovery. Our results suggest that targeting hormone‐related mechanisms could be a promising strategy for future diagnostic evaluation and therapeutic development for wound repair‐related diseases.

## 2 Results

### 2.1 Spatiotemporal Expression of THRA Upregulated on Post‐Wounding Day 3

Given that targeting hormone receptor activity directly influences cellular and molecular pathways, we began by examining the spatiotemporal expression of thyroid hormone receptors involved in the wound repair progress. Post‐Wounding Day 3 (PWD3) was selected as the initial time point as it marks the onset of the proliferative phase, characterized by granulation tissue formation and re‐epithelialization,^[^ ^3^ ^]^ which are central to our investigation of Thra activation dynamics. Single‐cell RNA sequencing (scRNA‐seq) analysis of homeostatic (HST) skin and PWD3 skin revealed a striking transcriptional induction of Thra, the gene encoding thyroid hormone receptor, transitioning from near‐undetectable levels in HST skin to robust expression at PWD3 (Figure 1B). Consistently, both bulk RNA‐sequencing and quantitative reverse transcription polymerase chain reaction (qRT‐PCR) results demonstrated a significant increase in Thra expression levels at the wound edge on PWD3 (Figure 1C). The western blotting analysis also supported this conclusion, showing elevated THRA protein levels on PWD3 (Figure 1D). We also compare the expression differences of Thra and thyroid hormone receptor type beta (Thrb)^[^ ^22^ ^]^ and found that the increase in Thra expression was significantly greater than that of Thrb when comparing HST and PWD3 skin (Figure S1A–F, Supporting Information). These results confirm that wounding could cause an increase in Thra levels near the wound area, suggesting that Thra may play a key role in skin wound healing. Furthermore, we performed targeted knockdown experiments using shRNA‐encoding lentiviruses (shThra, shThrb and shNC). Compared with the negative control groups, Thra knockdown group (shThra) significantly impaired epithelial regeneration. In contrast, Thrb knockdown group (shThrb) exhibited minimal impact (Figure S1G, Supporting Information). These results indicate that Thra, but not Thrb, plays a pivotal role in promoting epithelial regeneration.

To investigate the skin‐layer‐specific repair program mediated by Thra, we isolated epithelial (EPI) and fibroblast (FB) cell populations and observed an increase in Thra expression post‐wounding in both cell types (Figure 1E). Further spatial transcriptomic analysis revealed that the elevated Thra expression levels were mainly localized at the wound edge on PWD3 (Figure 1F). To obtain more spatiotemporal information, we tracked THRA expression across HST, PWD3, PWD5, and PWD7, with PWD5 and PWD7 chosen to capture the progression of the proliferative phase and the early remodeling phase.^[^ ^23^, ^24^ ^]^ This revealed a pattern in which THRA expression rapidly increased after injury, peaked around PWD3, and then declined during the wound repair process (Figure 1G,H). Also, the spatiotemporal expression of THRA is closely linked to that of PCNA, a marker commonly used to assess cell proliferation, further implying that Thra is crucial for wound repair and that its expression returns to homeostatic levels as the wound heals.

To clarify the layer‐specific regulatory roles of Thra in wound repair, we performed Gene Ontology (GO) enrichment analysis of Thra^+^ cells in EPI and FB post‐wounding, revealing significant enrichment of signals related to “response to wounding,” “cell development,” and “regulation of wounding” in the epidermis, as well as “extracellular structure organization” and “cell chemotaxis” in the dermis (Figure 1I). Meanwhile, these wounding‐specific signatures were entirely absent in steady‐state Thra^+^ populations (Figure S1H, Supporting Information), confirming their repair‐specific characteristics. Furthermore, enrichment analysis of all Thra‐positive cells in the wound data revealed a significant enrichment of signals related to the wound repair process (Figure S1I, Supporting Information). These findings suggest that Thra is essential for coordinating the wound repair process by regulating distinct functions in the epidermis and dermis.

### 2.2 Inhibition of Thra Delayed Epithelialization and Dermal Collagen Deposition

**Figure 2:**
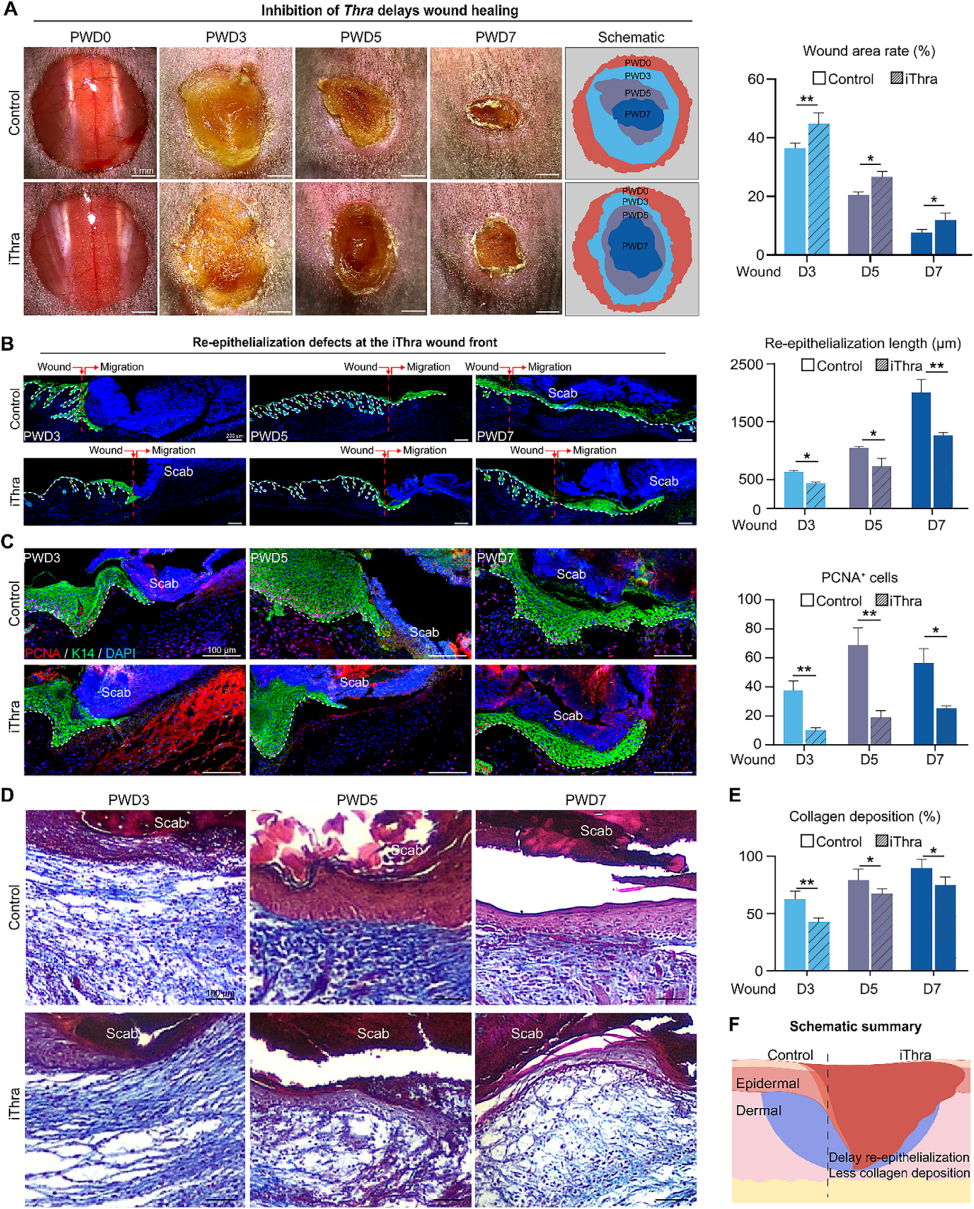
Inhibition of Thra delayed epithelialization and dermal collagen deposition. A) Phase‐contrast microscope images and schematic analysis of wound healing process after Thra inhibition treatment, with statistical analysis of the average wound rate. (Scale bars, 1 mm. N = 5, ^*^ ^*^p < 0.01, ^*^p < 0.05). B) Immunofluorescence images of K14 expression in the control and Thra inhibition groups, with statistical analysis of re‐epithelialization length. (Scale bars, 200 µm; N = 5, *p* <0.01, ^*^p < 0.05). C) Immunofluorescence images of K14/PCNA expression in the control and Thra inhibition groups, with statistical analysis of the average number of PCNA^+^ cells. (Scale bars, 100 µm; N = 5, ^*^ ^*^p < 0.01, ^*^p < 0.05). D) Masson's trichrome staining images of the control and Thra inhibition groups. (Scale bars, 100 µm). E) Statistics of the average collagen deposition. (N = 5, ^*^ ^*^p < 0.01, ^*^p < 0.05). F) Schematic summary of the wound healing process after Thra inhibition treatment.

Having established the spatiotemporal pattern of Thra, we confronted a pivotal question: does this evolutionary tuned expression drive functional repair? Subsequently, a small‐molecule inhibitor of Thra, SR35021,^[^ ^25^ ^]^ was injected around the wound site prior to injury, and wound repair in mice was observed on PWD3, PWD5, and PWD7 (Figure S2A–C, Supporting Information). Compared to the control group, inhibition of Thra led to a significantly reduced wound healing rate and an increased wound area in the skin of mice (**Figure** **2**A). Immunofluorescence staining revealed weaker re‐epithelialization in mice after Thra inhibition (Figure 2B). Statistical analysis demonstrated that the number of proliferating cells labeled by PCNA^+^ and P63^+^ was significantly reduced in the inhibitory group compared to the control group (Figure 2C; Figure S2D, Supporting Information). Furthermore, Masson's trichrome staining results demonstrated a significant decrease in collagen deposition and a more disorganized and uneven tissue structure following Thra inhibition (Figure 2D,E). This multimodal functionality—epithelial regeneration and stromal reinforcement—positions Thra as a pivotal regulator orchestrating the progressive adaptation of skin wound repair, with its inhibition disrupting the two essential foundations of the repair process (Figure 2F).

### 2.3 High Expression Levels of TH Promoted Wound Repair

**Figure 3:**
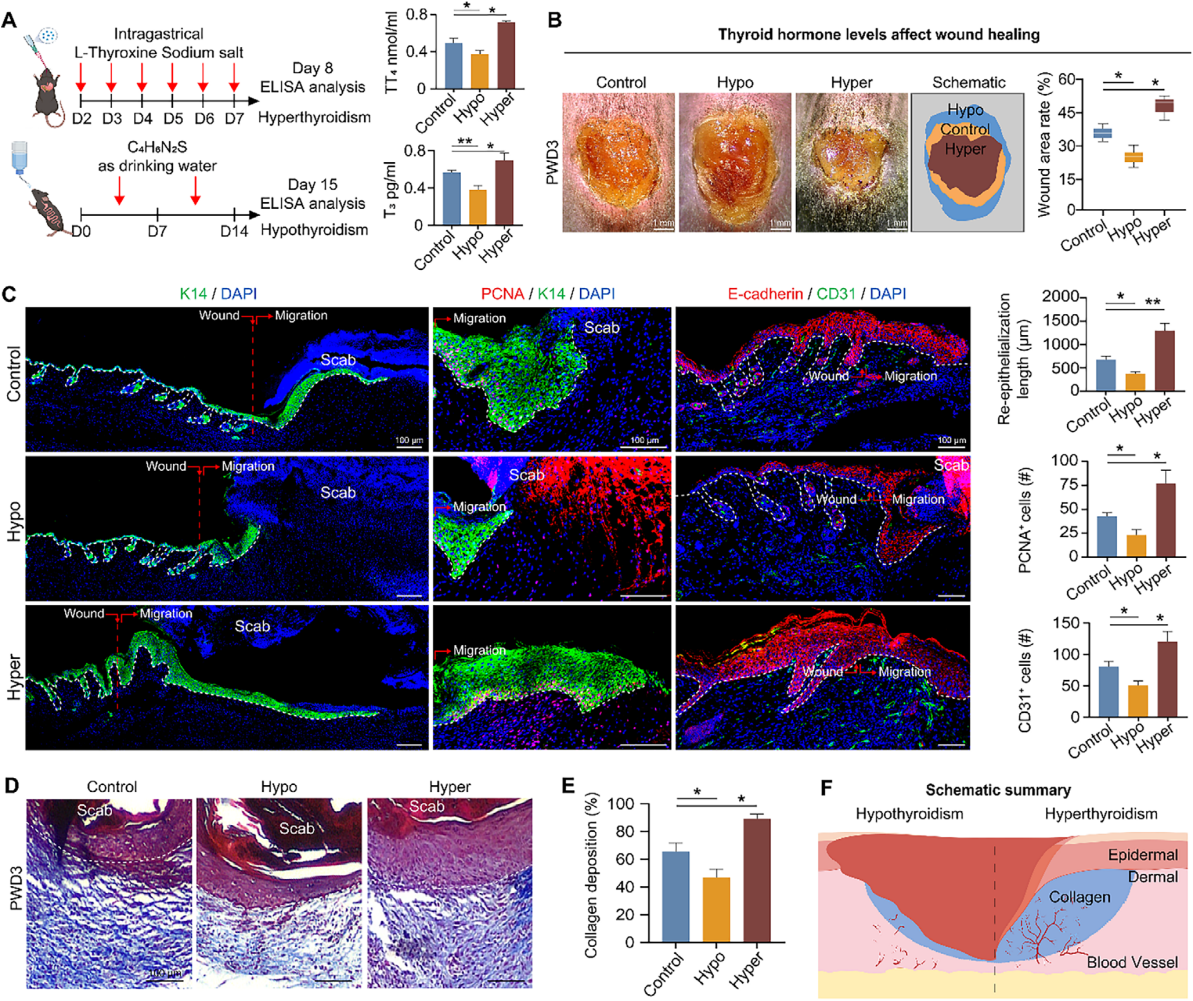
High expression level of TH promoted wound repair. A) Schematic illustration of the experimental design for hyperthyroidism and hypothyroidism models, with statistical analysis of average TT_4_ and T_3_ concentrations from ELISA assays. (N = 5, ^*^ ^*^p < 0.01, ^*^p < 0.05). B) Phase‐contrast microscope images and schematic analysis of wound healing status on PWD3 after thyroid dysfunction treatments, with statistical analysis of the average wound area rate. (Scale bars, 1 mm. N = 5, ^*^p < 0.05). C) Immunofluorescence images of K14, PCNA/K14 and E‐cadherin/CD31 expressions in the control, hypothyroidism and hyperthyroidism groups, with statistical analysis of the average re‐epithelialization length, and the average numbers of PCNA^+^ and CD31^+^ cells. (Scale bars, 100 µm; N = 5, ^*^ ^*^p < 0.01, ^*^p < 0.05). D) Masson's trichrome staining images of the control, hypothyroidism and hyperthyroidism groups. (Scale bars, 100 µm). E) Statistics of the average collagen deposition. (N = 5, ^*^p < 0.05). F) Schematic summary of the wound healing process after thyroid dysfunction treatments.

Having validated the essential role of Thra in wound repair, we next probed whether its ligand—thyroid hormone (TH)—serves as the upstream conductor of the observed Thra‐mediated regenerative process. It is known that Thra, as a receptor for TH, is essential in regulating cellular metabolism and function.^[^ ^26^, ^27^ ^]^ To revisit the impact of TH on wound repair, as initially intended, we established hyperthyroid and hypothyroid mouse models through oral administration of L‐thyroxine (T4) and methimazole, respectively. The alterations in TH levels in these mice were confirmed through enzyme‐linked immunosorbent assay (ELISA) results (**Figure** **3**A; Figure S3A, Supporting Information). By observing the wound repair progress in induced hyperthyroidism and hypothyroidism mouse models, we found that wound closure was fastest in the hyperthyroidism model, while the wound repair was significantly inhibited in the hypothyroidism model (Figure 3B; Figure S3B, Supporting Information). Immunofluorescence staining results on PWD3 showed that elevated TH levels promoted re‐epithelialization following injury, accompanied by a significant increase in the number of proliferating cells labeled by PCNA^+^ and P63^+^ in the epidermis of hyperthyroid mice, indicating an optimal proliferation status. Considering the release of TH originates from blood vessels and the remodeling of vascular structures is vital for wound healing,^[^ ^28^, ^29^ ^]^ we further investigated vascular regeneration. Immunofluorescence results on PWD3 revealed that mice with hyperthyroidism displayed the best vascular regeneration capacity with the highest CD31^+^ expression; while mice with hypothyroidism exhibited impaired vascular regeneration with the lowest CD31^+^ expression (Figure 3C; Figure S3C, Supporting Information). Masson's trichrome staining results showed that, compared to the control group, mice with hyperthyroidism displayed the densest dermal collagen deposition and the best collagen regeneration, whereas mice with hypothyroidism exhibited the poorest collagen deposition and irregular arrangement (Figure 3D,E). In conclusion, a high expression level of TH favors wound re‐epithelialization, vascular regeneration, and collagen deposition, thereby promoting wound repair (Figure 3F). These findings align with the observations from Thra inhibition experiments, providing evidence that TH triggers Thra‐mediated adaptive mechanisms in skin wound repair.

### 2.4 Thra‐Regulated Glutathione Metabolism in the Epidermis

**Figure 4:**
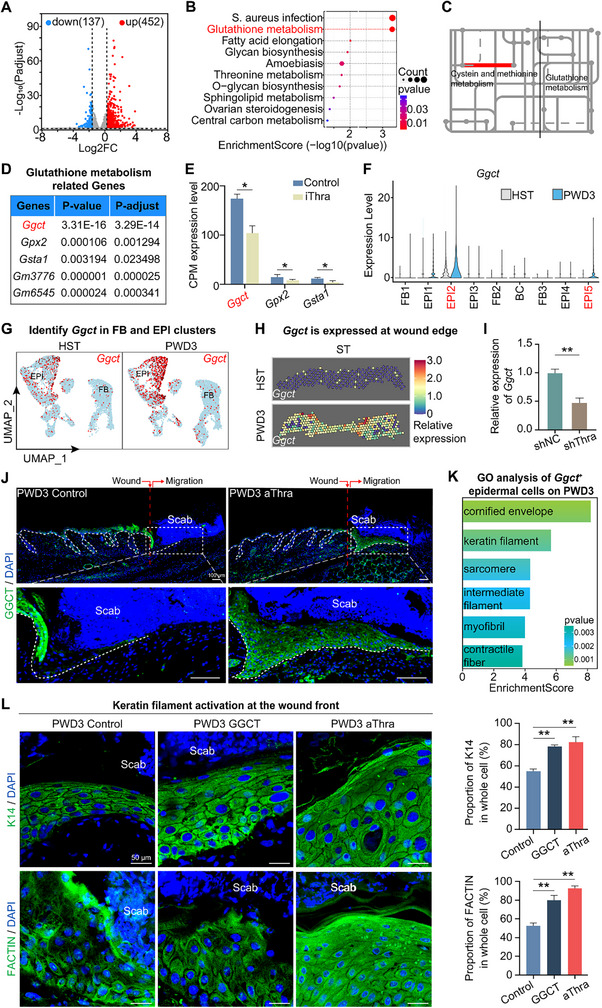
Thra‐regulated glutathione metabolism in the epidermis. A) Volcano Plot of the distribution of down‐ and up‐ regulated genes in the control and Thra‐inhibited wounded groups. B) KEGG analysis of pathway enriched in the Thra‐inhibited wounded group. C) iPATH analysis of the impact on metabolism pathways in the Thra‐inhibited wounded group. D) A list of the top 5 ranked genes related to glutathione metabolism according to their P‐values and p‐adjustments. E) Statistics of the expression levels of the top three ranked genes in the bulk RNA sequencing data in terms of CPM. (N = 3, ^*^p <0.05). F) VlnPlot of the expression of Ggct. G) FeaturePlots of the expression of Ggct in HST and PWD3 skin within FB and EPI clusters. H) Spatial transcriptomics data of the expression of Ggct in HST and PWD3 skin. I) qRT‐PCR analysis of Ggct expression levels after Thra knockdown. (N = 3, ^**^ *p* < 0.01) J) Immunofluorescence images of GGCT expression in the control and aThra groups. (Scale bars, 100 µm). K) GO analysis of pathways enriched in Ggct‐positive epidermal cells post‐wounding. L) Immunofluorescence images of K14 and FACTIN expressions in the control, GGCT and aThra groups, with statistical analysis of the average proportion of K14 and FACTIN in total cells. (Scale bars, 50 µm; N = 5, ^*^ ^*^p < 0.01).

To investigate the specific function of Thra, we conducted bulk RNA‐sequencing on control and Thra‐inhibited wounded skin (**Figure** **4**A), and then performed KEGG enrichment analysis on significantly downregulated genes identified. The analysis revealed that these genes were notably enriched in the glutathione metabolism pathway (Figure 4B). Additionally, iPATH analysis results also indicated the inhibition of the glutathione metabolism signaling pathway, leading to the activation of cysteine and methionine metabolism pathways, which was consistent with the results of differential gene enrichment (Figure 4C).

To delve deeper into the regulatory mechanisms of Thra on glutathione metabolism, we systematically ranked genes enriched in the glutathione metabolism pathway based on their p‐values and p‐adjustments (Figure 4D). We then examined the expression levels of the top three ranked genes in the bulk RNA sequencing data in terms of Counts Per Million (CPM), revealing that Ggct exhibited the highest expression level with statistical significance (Figure 4E). Meanwhile, we analyzed the expression of Ggct in single‐cell data from HST and wounded skin tissue. The findings indicated that Ggct displayed elevated expression levels in the epidermal cell population after wounding, whereas its expression in other cell populations was comparatively lower (Figure 4F). Featureplot analysis clearly depicted the expression patterns of Ggct in dermal and epidermal cells, further confirming this observation (Figure 4G). Accordingly, we hypothesized that Thra influence wound repair by regulating glutathione metabolism in the epidermis.

To validate this hypothesis, we examined the expression levels of key genes related to glutathione metabolism, namely Slc7a11, Gsr, and Gclm, in single‐cell data from hyperthyroidism and wounded skin. The results showed that genes related to glutathione metabolism were upregulated after wounding compared with the HST group (Figure S4A, Supporting Information). Results from immunofluorescence staining experiments demonstrated that mice receiving peri‐wound injections of glutathione (GSH) exhibited improved re‐epithelialization compared to the control group, with increased numbers of PCNA‐labeled and P63‐labeled cells, indicating enhanced proliferative capacity. Conversely, mice injected with an inhibitor of glutathione synthetase, BSO,^[^ ^30^ ^]^ showed suppressed re‐epithelialization, decreased numbers of PCNA‐labeled and P63‐labeled cells, and reduced proliferative capacity (Figure S4B,C, Supporting Information). These results demonstrated that Thra‐regulated glutathione metabolism could promote wound repair by accelerating re‐epithelialization, fulfilling the spatiotemporal demands required for a barrier‐first healing strategy.

### 2.5 GGCT Modulated Epidermal Cornified Envelope and Keratin Filament

Leveraging spatial transcriptomics data, we identified increased Ggct expression in the epidermal cells at the edges of the wound post‐injury (Figure 4H). Next, we packaged Thra‐targeting shRNA into lentivirus and delivered them into the back of wounded mice by subcutaneous injection. qRT‐PCR showed that knockdown (KD) of Thra inhibited the expression of Ggct compared with the non‐targeting control (NC)‐treated group (Figure 4I). Immunofluorescence staining validation demonstrated that GGCT was primarily expressed in the epidermis at the wound edge. After activating Thra at the wound edge, the area of GGCT expression is increased significantly (Figure 4J). In addition, injection of GGCT protein resulted in an increased number of P63‐labeled cells and enhanced proliferative capacity compared to the control group (Figure S4D, Supporting Information).

But how does this metabolic enzyme perform its specific function? We conducted Gene Ontology (GO) enrichment analysis on Ggct‐positive cells within the wounded epidermis, and the results revealed significant enrichment of pathways related to “cornified envelope” and “keratin filament” (Figure 4K). Further immunofluorescence staining results demonstrated elevated expression of K14, K16, and FACTIN in epidermal cells within the GGCT injection group and Thra activated group, while sparse FACTIN expression and compromised cellular scaffolds were observed in the control group (Figure 4L; Figure S4E, Supporting Information). Similarly, relative to the control group, the expression of K16 and FACTIN in epidermal cells was higher in the GSH injection group, while it was lower in the BSO injection group, accompanied by incomplete cellular scaffolds (Figure S4F, Supporting Information). Therefore, GGCT is a key factor in wound repair‐associated glutathione metabolism and, as a downstream target of Thra, could modulate cornified envelope and keratin filament dynamics, thereby influencing cellular mechanical support, maintaining epidermal cell architecture, and ultimately promoting epidermal epithelialization.

### 2.6 Thra‐Activated SAA3 Upregulated at the Dermal Wound Edge

**Figure 5:**
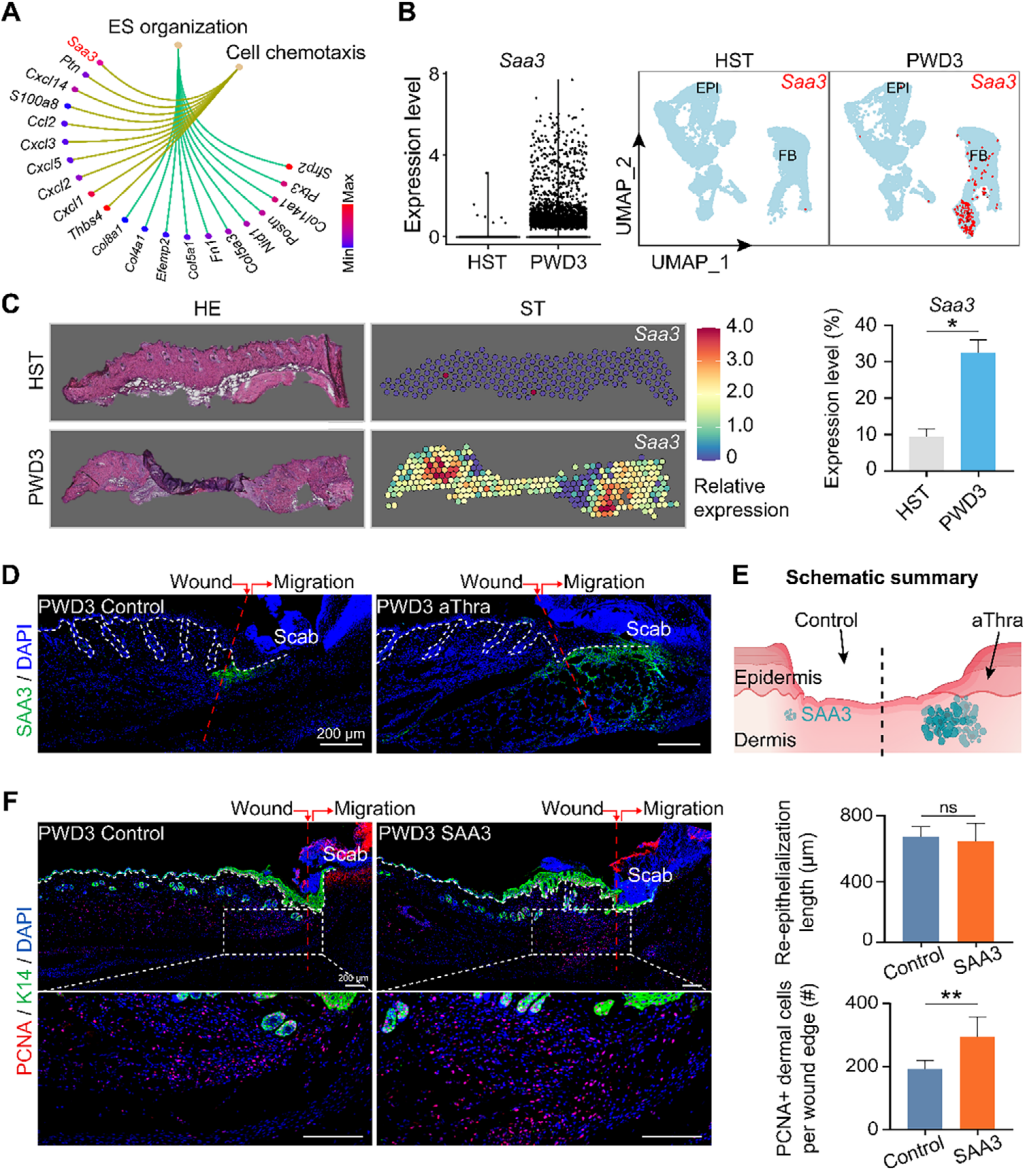
Thra‐activated SAA3 upregulated at the dermal wound edge. A) Pie chart of the genes in the top two signaling pathways in Thra‐positive cells in the FB post‐wounding. B) VlnPlot and FeaturePlots of the expression of Saa3. C) Spatial transcriptomics data of the expression of Saa3 in HST and PWD3 skin, with statistical analysis of expression level of Saa3. (N = 3, ^*^p < 0.05) D) Immunofluorescence images of SAA3 expression in the control and aThra groups. (Scale bars, 200 µm; N = 5) E) Schematic summary of the wound healing process after SAA3 treatment. F) Immunofluorescence images of PCNA/K14 expression in the control and SAA3 groups, with statistical analysis of the average re‐epithelialization length and the average number of PCNA^+^ cells. (Scale bars, 200 µm; N = 5, ^*^ ^*^p < 0.01, ns: no significance).

Thra's role in the epidermis, which fulfills the barrier‐first imperative, suggests an underexplored evolutionary rationale for its concurrent activity in the dermis, directing our attention to investigate its dermal function next. For the top two ranked signaling pathways and the genes within these pathways, we performed enrichment analysis on Thra‐positive FB cells post‐wounding (Figure 1I,A). We then selected the top four genes based on their ‐log_10_ (p‐value), namely Saa3, Cxcl1, Thbs4, and Sfrp2, and analyzed their expression patterns in single‐cell data from HST and wounded skin tissue. The results revealed that Saa3 exhibited the highest expression levels on PWD3 (**Figure** **5**B; Figure S5A, Supporting Information). Through featureplot visualization based on scRNA‐seq, we observed that Saa3 was highly expressed in dermal cells and scarcely expressed in epidermal cells (Figure 5B; Figure S5B,C, Supporting Information). Spatial transcriptomics data also indicated that while the expression of Saa3 was minimal in HST, it was significantly enhanced at the wound edge in the dermal region post‐wounding, whereas other genes were expressed at much lower levels and not related to specific locations (Figure 5C; Figure S5D, Supporting Information).

Based on these findings, we hypothesized that Thra in the dermis is associated with the activation of Saa3. Subsequently, we constructed lentiviral vectors carrying shRNA targeting Thra and transfected them into the mouse skin. qRT‐PCR analysis revealed that KD of Thra using shRNA significantly reduced the expression of Saa3 compared with the group treated with shNC (Figure S5E, Supporting Information). Immunofluorescence staining validation verified that the expression of SAA3 was increased in dermal cells beneath the wound tongue, and the expression level was even higher in the Thra‐activated group compared to the control group (Figure 5D,E). We also found that the number of PCNA^+^ proliferating cells in the dermis increased after injection of SAA3 protein, indicating enhanced proliferation of dermal cells (Figure 5F). Just as GGCT armored keratinocytes against mechanical stress, SAA3 architected the delayed yet precise matrix remodeling phase critical for long‐term structural integrity.

### 2.7 SAA3 Favored Dermal FN1 Protein Functions

**Figure 6:**
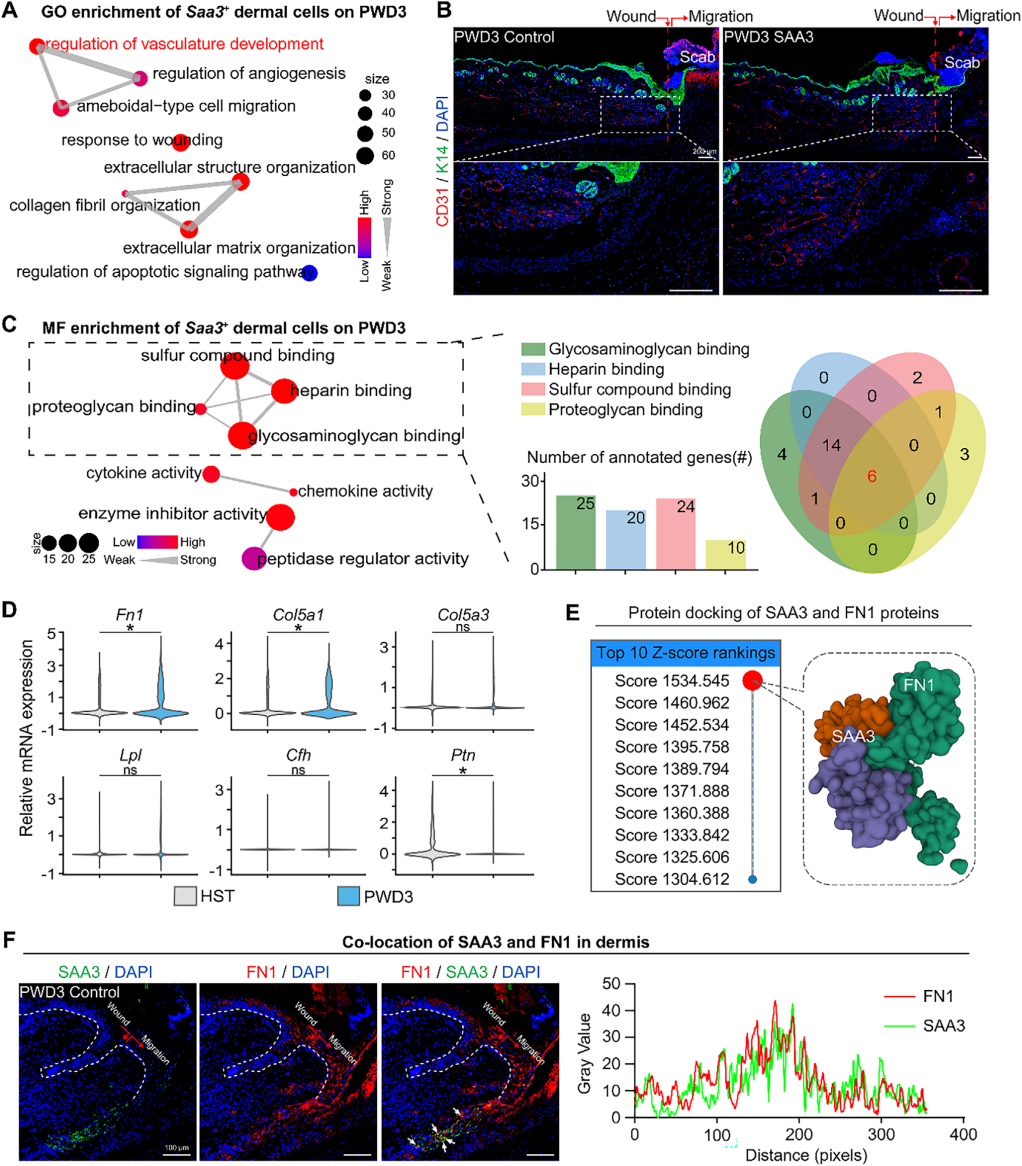
SAA3 favored dermal FN1 protein functions. A) GO analysis of pathways enriched in Saa3‐positive dermal cells post‐wounding. B) Immunofluorescence images of CD31/K14 expression in the control and SAA3 groups. (Scale bars, 200 µm; N = 5) C) MF analysis of pathways enriched in Saa3‐positive dermal cells post‐wounding; Venn Plot of the six most strongly related genes. D) VlnPlots of the expression of the six most strongly related genes. E) Protein docking of the binding status of SAA3 and FN1. F) Immunofluorescence images of SAA3, FN1 and FN1/SAA3 expressions post‐wounding. (Scale bars, 100 µm; Statistics of gray value traces).

To systematically explore Saa3's multifaceted functions in wound healing, we first confirmed its regulation by Thra and temporal upregulation post‐wounding. Intriguingly, when conducting Gene Ontology (GO) enrichment analysis on Saa3‐positive FB cells expressed after wounding, the results revealed significant enrichment of signaling pathways related to vascular development and angiogenesis regulation, such as “regulation of vasculature development” and “regulation of angiogenesis” (**Figure** **6**A). To validate this finding, we performed immunofluorescence staining to label blood vessels and found that compared to the control group, the group injected with SAA3 protein exhibited an increase in the number of CD31‐labeled blood vessels, showing better angiogenesis. Observed signs of vascular regeneration beneath the wound implied improved nutrient supply, waste clearance, and tissue regeneration (Figure 6B; Figure S6A, Supporting Information).

To further explore the molecular mechanism underlying SAA3 during wound healing, we conducted a Molecular Function (MF) enrichment analysis on Saa3‐positive FB cells expressed after wounding. The results revealed molecular interactions associated with binding to sulfide compounds, heparin, protein polysaccharides, and glycoproteins. The intersection of these pathways identified six highly relevant genes (Figure 6C), and analysis of single‐cell data from HST and wounded skin tissue showed upregulation of Fn1 and Col5a1 post‐wounding (Figure 6D). Additionally, we performed protein function enrichment analysis on these six genes, which indicated that FN1 occupied a central position in the protein network (Figure S6B, Supporting Information), suggesting its critical regulatory role in protein aggregation and important biological functions. Furthermore, using protein‐protein docking technology, we predicted the docking models of SAA3 with other proteins in a rigid docking manner. The results showed that the docking model score between SAA3 and FN1 was the highest, further confirming their interaction (Figure 6E; Figure S6C, Supporting Information). Based on these findings, we propose that SAA3, through its interaction with FN1, affects molecular functions such as binding to sulfide compounds, thereby regulating vascular development and regeneration during wound repair. FN1, as an extracellular matrix protein, also interacts with cell surface receptors to mediate cell signal transduction processes, playing a vital role in all stages of wound repair.^[^ ^31^, ^32^, ^33^, ^34^, ^35^ ^]^

### 2.8 GGCT, SAA3, and TH Regulatory Networks Demonstrated in Skin Organoid Models

**Figure 7:**
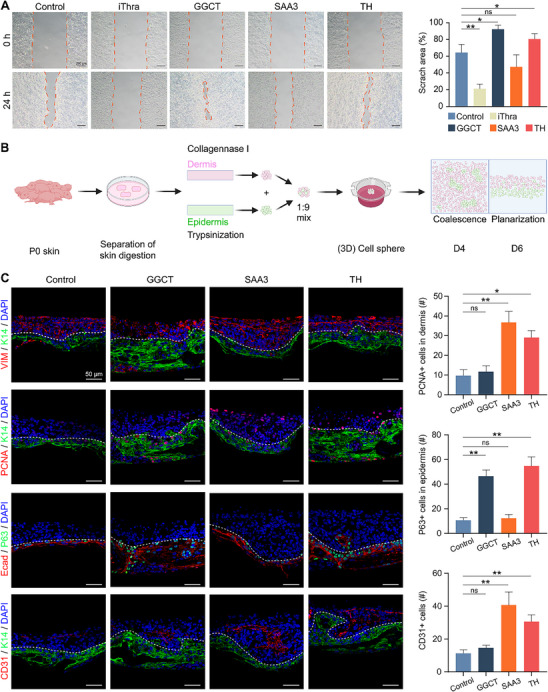
GGCT, SAA3, and TH regulatory networks demonstrated in mouse skin organoid models. A) Epithelial scratch assay results of the migratory ability of cells in different treatment groups, with statistical analysis of the average scratch area. (Scale bars, 200 µm; N = 3, ^*^ ^*^p < 0.01, ^*^p < 0.05, ns: no significance) B) Schematic illustration of the experimental design for mouse skin organoids. C) Immunofluorescence images of VIM/K14, PCNA/K14, Ecad/P63, and CD31/K14 expressions on the sixth day of cultured skin organoids, with statistical analysis of the average number of PCNA^+^ cells in dermis, the average number of P63^+^ cells in epidermis and the average number of CD31^+^ cells. (Scale bars, 50 µm; N = 3, ^*^ ^*^p < 0.01, ^*^p < 0.05, ns: no significance).

To corroborate the above findings of TH regulatory networks, we employed an in vitro epithelial scratch assay—a model reflecting the migratory dynamics critical to re‐epithelialization.^[^ ^36^ ^]^ Compared to the control group, Thra inhibition markedly impaired epithelial cell migration and delayed closure of the scratch wound. Conversely, supplementation of GGCT or TH enhanced migratory capability and accelerated wound healing, whereas SAA3 showed no significant effects (**Figure** **7**A). These results further suggest the role of Thra‐regulated glutathione metabolism, mediated by GGCT and TH, in promoting epithelial cell migration as a necessary component of a barrier‐first healing strategy.

To gain a more comprehensive understanding of the roles of GGCT, SAA3, and TH in wound repair, we further utilized a mouse skin organoid model for research. Skin organoids overcome the limitations of 2D cultures by modeling the 3D organization and multicellular interactions of native skin, allowing for a more precise representation of skin physiological processes.^[^ ^37^, ^38^ ^]^ Previous studies have found that during the self‐assembly process of organoids, cells re‐establish connections through interactions and signal transmission to form an orderly tissue structure,^[^ ^39^, ^40^ ^]^ including the spontaneous aggregation and self‐assembly of dermal and epidermal cells, ultimately achieving planarization (Figure 7B). This process shares similarities with cell proliferation, migration, and tissue reconstruction during wound repair.^[^ ^41^ ^]^

In mouse skin organoids, treatment with GGCT or TH enhanced epidermal cell proliferation, marked by larger aggregates and increased PCNA^+^ and P63^+^ cells at day 4, a trend sustained through day 6 (Figure S7A,C, Supporting Information). Concurrently, SAA3 or TH promoted vascular remodeling, evidenced by elevated CD31^+^ endothelial cell density and organization around dermal compartments at both time points (Figure S7A,C, Supporting Information). Given the superior physiological relevance of human skin organoids, we further explored the effects of these treatments in this model system. Human skin organoids offer several advantages, including a more accurate representation of human skin biology and enhanced translational potential for clinical applications. After culturing human skin organoids for 20 days (Figure S7B, Supporting Information), we performed micro‐injury using a 100 µm‐diameter microneedle to mimic skin damage (Figure S7C, Supporting Information). The results showed that treatment with TH significantly increased the size of human skin organoids and accelerated recovery from micro‐injury compared to the control group (Figure S7D, Supporting Information). These findings collectively demonstrate that GGCT and TH drive epidermal proliferation and self‐assembly, while SAA3 and TH synergistically enhance stromal reinforcement. By coordinating both epidermal re‐establishment and vascular niche development, TH‐induced regulatory networks ensure the spatiotemporal coupling of barrier restoration and tissue remodeling while facilitating epithelial‐stromal crosstalk and streamlining the repair cascade.

## 3 Discussion

As a paradigm of spatiotemporal adaptation in multicellular organisms, skin wound healing demonstrates how layered tissues mount rapid survival responses through temporally sequenced and spatially partitioned repair programs. This biological process is highly orchestrated, involving coordinated interactions of multiple cell types, signaling pathways, and molecular factors.^[^ ^42^ ^]^ In this study, we aimed to elucidate the roles of thyroid hormone and its receptor, Thra, in spatiotemporally coordinated wound repair, with a focus on their layer‐specific functions within the epidermis and dermis. Through a detailed analysis of Thra's spatiotemporal expression patterns and its spatiotemporally regulatory mechanisms during wound healing, we uncovered its critical importance in orchestrating this dynamic process.

Our findings demonstrated a significant spatiotemporal upregulation of Thra on PWD3, particularly in dermal and epidermal cells at the wound edge. It is well established that the nervous system is among the first responders to injury, initiating wound healing through pain perception, sensory signal transmission, and modulation of muscle activity.^[^ ^43^, ^44^ ^]^ Previous studies have shown that TH can promote regeneration in the central nervous system and support phase‐specific repair in the peripheral nervous system.^[^ ^45^, ^46^, ^47^ ^]^ Following skin injury, nerve impulses generated by the nervous system trigger increased TH secretion, which in turn creates a favorable environment for accelerated neural regeneration.^[^ ^48^ ^]^ This suggests that elevated thyroid hormone levels could be harnessed to promote wound healing in both the epidermis and dermis. As we mentioned before, the rupture and vasodilation of injured blood vessels can also facilitate the diffusion of TH from the bloodstream to the wound microenvironment. Pain signals can also increase vascular permeability, allowing TH and other wound repair factors to more easily reach the injury site to promote healing.^[^ ^43^, ^49^ ^]^ Moreover, previous studies have shown that increased TH levels can lead to the activation of Thra.^[^ ^50^ ^]^ As a nuclear receptor, Thra can form complexes with transcription factors and regulate the transcription of downstream genes. This process influences skin cell behaviors, including cell growth, differentiation, metabolism, immunity, and many other important aspects.^[^ ^51^ ^]^ These restored cellular behaviors, in turn, contribute to maintaining endocrine homeostasis.^[^ ^52^ ^]^

The spatiotemporal adaptation of epidermal repair is fundamentally driven by the imperative for rapid barrier restoration, where temporal urgency overrides spatial precision during initial healing phases.^[^ ^3^ ^]^ Before our investigation into the phase‐specific role of Thra in the epidermis following injury, previous studies had already established the importance of thyroid hormone receptors in epidermal homeostasis. For instance, knockout (KO) mice lacking thyroid hormone receptors exhibited reduced keratinocyte proliferation in the interfollicular epidermis.^[^ ^53^ ^]^ Our findings align with these observations, as we identified Ggct—a downstream target of Thra—as a regulator of epidermal epithelialization. This supports the notion that Thra is essential for maintaining epidermal function. Notably, GGCT is a key enzyme in the glutathione metabolism pathway.^[^ ^54^, ^55^ ^]^ Our prior research has shown that metabolic processes, including glutathione metabolism, are intricately linked to the health and function of epidermal cells. Specifically, alterations in metabolic pathways involving glutathione can profoundly influence epidermal cell behavior, including proliferation rates and cytoskeletal structure and function.^[^ ^56^ ^]^ Therefore, by connecting the dots between GGCT, glutathione metabolism, and the regulation of epidermal cell behavior, we can infer that Thra, through its downstream target Ggct, modulates epidermal function by influencing the metabolic landscape of the epidermis. This metabolic regulation could, in turn, affect the proliferation and overall health of epidermal cells, thereby maintaining the integrity and homeostasis of the skin.

In contrast, dermal spatiotemporal adaptation requires balanced coordination between structural support and phased architectural remodeling, demanding both spatial precision and temporal persistence. Regarding the role of Thra in the dermis, previous research has highlighted that thyroid hormones finely regulate intracellular glucose and lipid metabolism as well as systemic metabolic homeostasis through the TRβ receptor in adipocytes, which are primarily located beneath the dermis.^[^ ^57^ ^]^ Previous work revealed the critical role of thyroid hormones in systemic metabolism, particularly in white adipose tissue, where TRβ mediates their effects on multiple metabolic pathways by targeting ChREBP.^[^ ^57^ ^]^ Considering that tissue repair is a complex process involving rapid cell proliferation and the formation of new tissue, both of which require a substantial amount of energy and metabolic substrates to proceed through the whole thick skin layers.^[^ ^58^ ^]^ Taking our findings together, the role of Thra in wound repair is multifaceted; it not only regulates dermal cell proliferation and vasculature development but also provides structure and nourishment to the overlying epidermis, making it an indispensable part of the wound healing process.

It is worth mentioning that we identified a significant population of cells expressing Saa3 in injured tissues. Prior studies have indicated that SAA3 may interact with specific cell receptors, thereby stimulating the production of collagenase (matrix metalloproteinase‐1) in rabbit dermal fibroblasts.^[^ ^59^ ^]^ This aligns with our observation that SAA3 plays an important role in promoting dermal repair. Moreover, studies have suggested that SAA3 expression markedly increases during the differentiation process in 3T3‐L1 adipocytes stimulated by corticosterone and interleukin‐6.^[^ ^60^ ^]^ We note that SAA3 activation is not only regulated by Thra and may be closely associated to multiple hormones governed by our endocrine system. By further exploring the mechanisms of action of dermal cells expressing the Saa3 gene under various hormone profiles, we are expected to provide a deeper understanding and new therapeutic targets for future treatment strategies.

**Figure 8:**
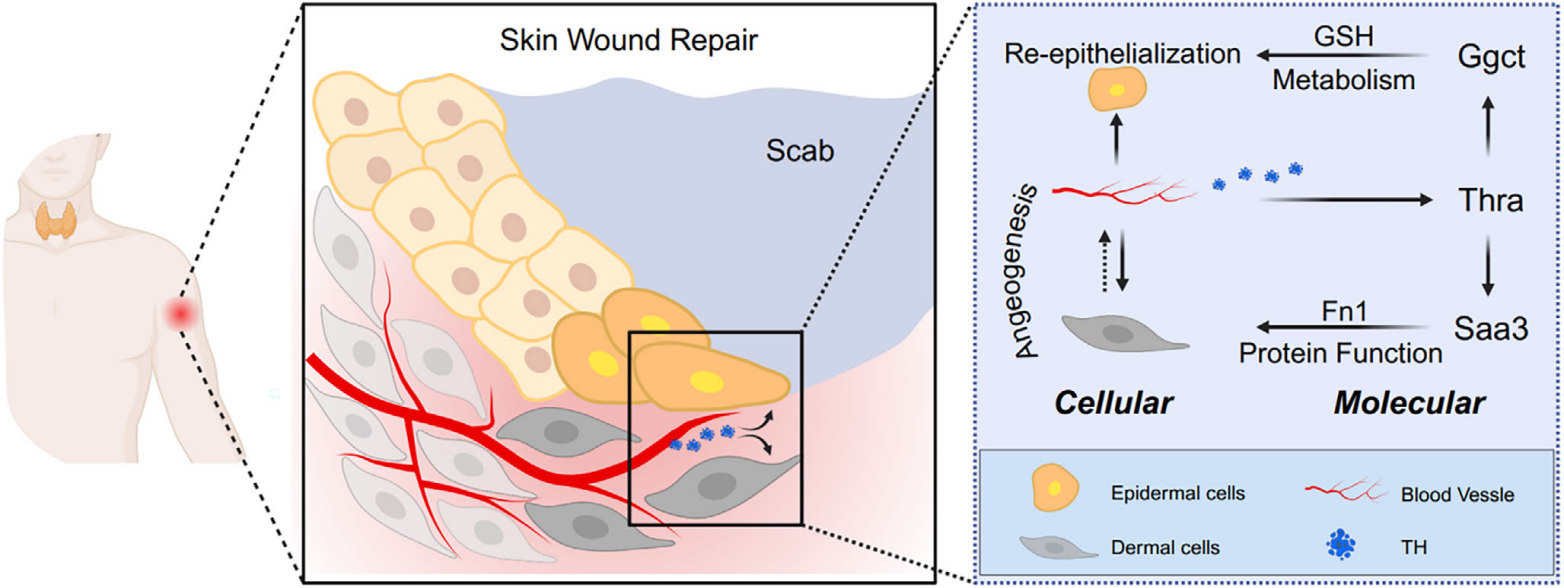
Graphical abstract. The activation of Thra in the epidermis influenced the expression of GGCT, regulating glutathione metabolism in epidermal cells and promoting epithelial regeneration. In the dermis, Thra upregulated SAA3 expression and interacted with FN1, regulating vascular development and supporting molecular functions.

In summary, our study has characterized the spatiotemporal expression patterns of Thra post‐wounding, and delved into the mechanisms of Thra in the epidermis and dermis respectively, providing a comprehensive evaluation of its phase‐coupled role in wound repair (**Figure** **8**). These findings provide a comprehensive evaluation of Thra's role in spatiotemporally stratified wound repair and offer insights into how the endocrine system influences this process through hormone release, hormone‐induced metabolism, and hormone‐regulated protein functions. To validate the functionality of key molecular targets, including TH, Thra, Ggct, and Saa3, we employed multiple models, such as wounded mice, hyperthyroidism/hypothyroidism mice, and skin organoids, all of which yielded consistent results. While our multi‐model approaches robustly support the spatiotemporal roles of Thra, each model offers distinct insights. The wounded mouse model captures physiological Thra activation in vivo, reflecting the natural wound environment. Hyperthyroid and hypothyroid models allow controlled modulation of TH‐Thra signaling through systemic endocrine fluctuations. Skin organoids, albeit lacking systemic hormonal regulation, isolate epidermal‐dermal crosstalk and validate their involvement in the process of skin wound healing. Collectively, our work not only advances the understanding of wound repair mechanisms but also provides valuable insights for developing novel treatments and improving patient care.

## 4 Experimental Section

### Ethics Statement

The experimental protocol was approved by the Institutional Animal Care and Use Committees of Chongqing University (Approval No: COCH‐LAE‐A0000202055). Written informed consent was obtained from all participants. This project was conducted at the Animal Experiment Center of Chongqing University Affiliated Tumor Hospital. The environmental conditions of the facilities complied with standards outlined in the Chinese National Standard “Laboratory Animal Environment and Facilities” (GB14925‐2010) for conventional/barrier animal experimental facilities. All animal housing and experimental procedures adhered to the requirements of the “Chongqing Municipal Experimental Animal Management Regulations” (Yufu Order No. 195) and other relevant regulations. Professional laboratory instructors were available for guidance and supervision throughout the study, ensuring compliance with the regulatory requirements for the proposed animal experiments.

### Enzyme‐Linked Immunosorbent Assay

At room temperature, the blood was allowed to coagulate naturally for 10–20 min, followed by centrifugation at 2000–3000 rpm for 20 min. The resulting supernatant was collected, and if any precipitation occurred during storage, the sample underwent additional centrifugation. Subsequently, 50 µL of the standard solution was added to the designated wells, while 40 µL of sample diluent was added to the sample wells, along with 10 µL of the test sample. After mixing thoroughly, 100 µL of enzyme‐labeled reagent was added to all wells. The plate was then sealed and incubated at 37 °C for 60 min, followed by washing five times with wash buffer, each for 30 s. Next, 100 µL of chromogenic substrate was added to each well, and incubated at 37 °C in the dark for 15 min. Finally, 50 µL of stop solution was added to terminate the reaction. After zeroing with blank wells, the Absorbance (OD values) of each well was measured sequentially at 450 nm wavelength (Molecular Devices, China). All the reagents mentioned above were obtained from a kit (Coibo, China).

### Hyperthyroidism/Hypothyroidism Mice

C57BL/6 mice were used in this study (Gempharmatech, China). The hyperthyroidism mice were treated with a suspension of 0.02 g mL^−1^ levothyroxine sodium tablets (Merck, Germany), via a gastric gavage at 0.02mL kg^−1^ once daily for 7 consecutive days. The hypothyroidism mice were provided with drinking water containing 0.04% methimazole (#M106466, Aladdin, China) for 14 days.

### Mouse Skin Organoid Culture

CD1 mice were used in this study (Gempharmatech, China). The primary culture of newborn mouse cells was conducted following the protocol outlined in our previous publications.^[^ ^37^, ^41^ ^]^ In brief, cells were isolated from the dorsal skin of newborn mice within 24 h of birth. The dorsal skin was floated overnight in a 0.25% trypsin solution (#15050057, Gibco, USA) at 4 °C to separate the dermis and epidermis. Epidermal cells were obtained by cutting, filtration, and centrifugation. Dermal cells were digested in 0.35% collagenase I (#LS004197, Worthington, USA) for 20 min, followed by filtration and centrifugation. The dissociated epidermal and dermal cells were then mixed at a 1:9 ratio and seeded in the upper chamber of a Transwell culture system, with the lower chamber containing 700 uL of DMEM/F12 culture medium (#MT10013CV, Corning, USA) supplemented with 10% FBS (#10099‐141C, Gibco, USA). Cultures were maintained in a 5% CO_2_ incubator at 37 °C, with medium renewal every other day.

### Human Skin Organoid Culture

Induced pluripotent stem cells (hPSC line derived from PBMCs) were dissociated into single cells and seeded into 96‐well U‐bottom plates to create uniform cell aggregates. The aggregates were then transferred to new plates containing differentiation medium to promote epidermal differentiation as previously described.^[^ ^61^ ^]^ Organoid cultures were maintained in a 37 °C incubator with 5% CO_2_, and the medium was refreshed every two days.

### Western Blot

Protein samples were separated by SDS‐PAGE electrophoresis and transferred onto a polyvinylidene fluoride (PVDF) membrane (#ISEQ00010, Millipore, USA). The membrane was blocked with 5% non‐fat milk solution at room temperature for 1 h, then washed twice with Tris‐buffered saline containing Tween (TBST)(#BL315B, Biosharp, China)for 5 min each. It was then incubated overnight at 4 °C on a shaker with specific primary antibodies. Subsequently, the membrane was washed four times with TBST, 5 min each. Afterward, it was incubated at 37 °C for 60 min with secondary antibodies, followed by four washes with TBST for 5 min each. Finally, visualization was achieved using an enhanced chemiluminescence (ECL) substrate (Bio‐OI, China), and the results were analyzed using ImageJ software.

### Transcriptome Analysis

Eukaryotic mRNA sequencing involved extracting total RNA from tissue samples, Library construction and sequencing were performed at Shanghai Majorbio Bio‐pharm Biotechnology Co., Ltd. (Shanghai, China) according to the manufacturer's instructions (Illumina, San Diego, CA). Bulk RNA‐seq data were analyzed on the Majorbio Cloud Platform (https://www.majorbio.com). And single‐cell data of unwound and wounded mice were downloaded from the gene ontology (GEO) database (GSE166948). The data were reanalyzed using R and Rstudio with Seurat v.4.1.1 functions. The Read10X function loaded 10x Genomics data, followed by cell QC to remove low‐quality cells. Data were normalized, underwent feature selection, linear and non‐linear dimensional reduction, cell clustering, and the finding of cluster biomarkers, and cell type identity was assigned to clusters. Visualization plots were generated for clustering results and cell subgroups, followed by functional enrichment analysis using clusterProfiler, enrich KEGG, and org.Hs.eg.db for GO and KEGG analysis. The spatial transcriptome data of homeostatic and wounded mouse skin were downloaded from the literature.^[^ ^36^ ^]^ The ‘hdf5r’ function was used for data reading and writing in HDF5 format. Data standardization was done by the SCTransform function to correct biases and heterogeneity. The SpatialFeaturePlot function visualized the spatial distribution of target genes.

### Real‐Time Quantitative Reverse Transcription PCR (qRT‐PCR)

Tissue samples were ground using a mortar and pestle, and total RNA was extracted using Trizol reagent (#NR0002, Leagene, China). The extracted RNA was converted into cDNA using the ReverTra Ace RT‐qPCR kit (#RR047Q, Takara, Japan) following the manufacturer's instructions. Quantitative PCR analysis was conducted on a real‐time fluorescence quantitative PCR instrument (LightCycler480, Roche, German) using SYBR Green PCR Master Mix (#RR820A, Takara, Japan); Gapdh was used as an internal control. The fluorescence signals were analyzed using the software of the qPCR instrument, and the CT values for the target gene were calculated. The relative expression levels of the target genes in each sample were calculated by normalizing the CT values to those of the internal reference gene. Primer sequences used are as follows: Thra (Forward: 5’ GAACAGCTCAAGAATGGTGGC 3’; Reverse: 5’ GAATCGAACTCTGCACTTCTCTC 3’), GAPDH (Forward: 5’ AGGTCGGTGTGAACGGATTTG 3’; Reverse: 5’ TGTAGACCATGTAGTTGAGGTCA 3’), Ggct (Forward: 5’ GGGGAGACATTCCTGTACTTCG 3’; Reverse: 5’ TTTTGCCTTGGAAATTGCCGA 3’), Saa3 (Forward: 5’ TGCCATCATTCTTTGCATCTTGA 3’; Reverse: 5’ CCGTGAACTTCTGAACAGCCT 3’).

### Small Molecules Perturbation

For in vivo experiments, if the samples were collected on PWD3, recombinant proteins, and small molecule drugs were injected subcutaneously on PWD −1, 0, and 2, respectively. If the samples were harvested on PWD5, the drugs were subcutaneously injected on PWD −1, 0, 2, and 3, respectively. If the samples were collected on PWD7, the drugs were subcutaneously injected on PWD −1, 0, 2, 3, and 5. The injection schematic is depicted in Figure S2 (Supporting Information). For in vitro experiments, small molecules or recombinant proteins were added to the culture medium at varying concentrations for treatment.

### Immunofluorescence Staining

The processed samples were fixed in a 4% paraformaldehyde (PFA) solution (#BL539A, Biosharp, China) at 4 °C for 48 h. After fixation, the PFA was washed away, and the samples were permeabilized using PBS containing 0.1% Triton X‐100 (#T8200, Solarbio, China). Subsequently, the samples were dehydrated, embedded, and cut into 6–8 µm sections. Dewaxing was performed at 65 °C, followed by gradual rehydration. Antigen retrieval was carried out using citric acid (#C805019, Macklin, China) and sodium citrate (#S818273, Macklin, China) solutions. The sections were blocked with 1% bovine serum albumin (BSA; #A8020, Solarbio, China) buffer at 37 °C for 60 min, and then incubated overnight with appropriate primary antibodies at 4 °C. The next day, the sections were washed three times with PBS and incubated with the corresponding fluorescently labeled secondary antibodies at 37 °C for 2 h to visualize the target molecules. DAPI (4'6'‐diamidino‐2‐phenylindole) (#C0065, Solarbio, China) was used for counterstaining to label the cell nuclei. Finally, the slides were sealed with an anti‐fade mounting medium (#S2100, Solarbio, China) and stored at 4 °C. The following antibodies were used in the study: K14 (Boster, BM4522), VIM (BEYOTIME, AF0318), PCNA (Elabscience, E‐AB‐22001), CD31 (Servicebio, GB12063‐100), P63 (Zenbio, 381215), Thra (Affinit, AF9218), E‐cadherin (Beyotime, AF0138), GGCT (Abcam, ab198503), K16 (Affinity, AF5482), FACTIN (Invitrogen, A12379), SAA3 (Abcam, ab282730), FN1 (Santa, sc‐8422). All primary antibodies were diluted at 1:150, and the secondary antibody at 1:500.

### Lentiviral shRNA Knockdown

shRNA sequences targeting Thra and Thrb genes were designed, synthesized, and cloned into the pLKO.1‐EGFP‐puro vector via enzymatic digestion and ligation reactions. The correct recombinant plasmids were obtained after sequence verification. Lentivirus packaging was performed using a standard three‐plasmid system, including the recombinant lentiviral expression plasmid, psPAX2, and pMD2.G, which were co‐transfected into 293T cells. After collecting the cell supernatant, viral particles were concentrated by ultracentrifugation to obtain high‐titer lentivirus concentrate, which was aliquoted and stored at −80 °C. For cell transfection, target cells were cultured to an appropriate density and transfected with the lentivirus concentrate. Transfection efficiency was assessed by EGFP expression under a fluorescence microscope (Figure S8A, Supporting Information). The shRNAs exhibiting the highest knockdown (KD) efficiency were selected for subsequent experiments. For in vivo delivery, 1 × 10^7^ PFU of lentiviral particles (shThra, shThrb, shNC) in 50 µL were injected subcutaneously into the exposed dorsal skin 2 days before and on the day of wounding. Samples were collected and analyzed on PWD3. The shRNA sequences are listed in Figure S8B (Supporting Information).

### Proteins Analysis Techniques

The protein list (without applying a cutoff value) was submitted to the STRING database (https://string‐db.org/), selected Mus musculus as the study organism, and used the “proteins with values/ranks” mode to rank the proteins for further analysis. Rigid protein‐protein docking (ZDOCK) was performed between SAA3 and FN1 to examine their interactions. Protein structural domains in PDB format were obtained from the Protein Data Bank (PDB) database (http://www.rcsb.org/). The ZDOCK module was used to identify docking sites and calculate ZDOCK scores.

### Masson's Trichrome Staining

Tissue sections were immersed in staining solution overnight at room temperature and then rinsed with running water for 10 min. They were then stained with methylene blue dye for 2–3 min, followed by two washes in distilled water. Next, the sections were stained with Mayer's hematoxylin dye for 2–3 min and rinsed twice with distilled water. The samples were briefly differentiated in an acidic differentiation solution for 2–5 min, rinsed with water to stop differentiation, and then washed again with distilled water. They were then stained with eosin Y stain for 10 min and rinsed twice with distilled water. After processing the samples for ≈10 min and discarding the upper layer solution, they were stained with aniline blue dye for 5 min. An acidic solution was used to wash away the aniline blue dye, followed by covering the sections with a weak acid working solution for 2 min. Subsequently, dehydration was performed using 95% ethanol for 30 s, followed by two rounds of dehydration in absolute ethanol, first for 30 s and then for 1 min. Finally, the sections were cleared twice with xylene for 1–2 min each before mounting with neutral gum. All the reagents mentioned above were obtained from a kit (#G1340, Solarbio, China).

### Statistical Analyses

Group sizes were determined on the basis of the results of the preliminary experiment and mice were assigned at random to groups. The number of animals shown in each figure is indicated in the legends as n = x mice per group and in times, and data are presented with multiple measurements per animal. Data are presented as the mean value ± standard error of the mean (SEM), calculated from no fewer than three separate experiments. A two‐tailed, unpaired Student's t‐test was employed to evaluate the differences in sample means. A one‐way analysis of variance (ANOVA) was utilized to examine the disparities among the means of different groups. In instances where ANOVA revealed a significant effect, indicating that at least one group's mean was distinct from the others, Tukey's honestly significant difference (HSD) test was subsequently applied for post hoc analysis. A p‐value less than 0.05 was deemed to represent statistical significance. The entirety of the statistical analyses was carried out utilizing GraphPad Prism 8 software.

## Conflict of Interest

The authors declare no conflict of interest.

## Author Contributions

Z.L. contributed to conceptualization, methodology, data curation, investigation, and original draft writing. J.T., C.Z., S.Z., Y.Y., and X.L. were involved in validation, data curation, and visualization. X.X., Y.Z., and J.W. participated in original draft writing, review, editing, and funding acquisition. X.S., T.X., M.W., J.J., and Y.Z. contributed to methodology and visualization. M.L. and C.‐M.C. oversaw conceptualization, supervision, manuscript review and editing, project administration, and funding acquisition.

## Supporting information



Supporting Information

## Data Availability

Research data are not shared.

## References

[1] S. Ben‐Moshe , T. Veg , R. Manco , S. Dan , D. Papinutti , A. Lifshitz , A. A. Kolodziejczyk , K. Bahar Halpern , E. Elinav , S. Itzkovitz , Cell Stem Cell 2022, 29, 973. 35659879 10.1016/j.stem.2022.04.008

[2] F. Chen , J. S. Tchorz , Cell Stem Cell 2022, 29, 871. 35659871 10.1016/j.stem.2022.05.008

[3] O. A. Peña , P. Martin , Nat. Rev. Mol. Cell Biol. 2024, 25, 599. 38528155 10.1038/s41580-024-00715-1

[4] X. Sun , S. Joost , M. Kasper , Cold Spring Harb. Perspect. Biol. 2023, 15, a041232. 36376081 10.1101/cshperspect.a041232 PMC10153802

[5] H. E. Talbott , S. Mascharak , M. Griffin , D. C. Wan , M. T. Longaker , Cell Stem Cell 2022, 29, 1161. 35931028 10.1016/j.stem.2022.07.006 PMC9357250

[6] S. Sinha , H. D. Sparks , E. Labit , H. N. Robbins , K. Gowing , A. Jaffer , E. Kutluberk , R. Arora , M. S. B. Raredon , L. Cao , S. Swanson , P. Jiang , O. Hee , H. Pope , M. Workentine , K. Todkar , N. Sharma , S. Bharadia , K. Chockalingam , L. G. N. de Almeida , M. Adam , L. Niklason , S. S. Potter , A. W. Seifert , A. Dufour , V. Gabriel , N. L. Rosin , R. Stewart , G. Muench , R. McCorkell , et al., Cell 2022, 185, 4717. 36493752 10.1016/j.cell.2022.11.004 PMC9888357

[7] Z. Li , R. Ma , J. Tan , C. Li , Y. Xiao , X. Qiu , S. Jin , P. Ouyang , Y. Zhao , X. Xiang , W. Wu , Mol. Med. 2024, 30, 217. 39543465 10.1186/s10020-024-00978-6 PMC11566089

[8] H. Kiaris , I. Chatzistamou , A. G. Papavassiliou , A. V. Schally , Trends Endocrinol. Metab. 2011, 22, 311. 21530304 10.1016/j.tem.2011.03.006

[9] L. Du , B. M. Ho , L. Zhou , Y. W. Y. Yip , J. N. He , Y. Wei , C. C. Tham , S. O. Chan , A. V. Schally , C. P. Pang , J. Li , W. K. Chu , Nat. Commun. 2023, 14, 3298. 37280225 10.1038/s41467-023-39023-1 PMC10244428

[10] A. Esposito , L. Ambrosino , S. Piazza , S. D'Aniello , M. L. Chiusano , A. Locascio , Cells 2021, 10, 3391. 34943899 10.3390/cells10123391 PMC8699336

[11] A. Wang , Y. Liu , Y. Yan , Y. Jiang , S. Shi , J. Wang , K. Qiao , L. Yang , S. Wang , S. Li , W. Gui , Environ. Sci. Technol. 2025, 59, 142. 39718545 10.1021/acs.est.4c07890

[12] G. A. Brent , Thyroid 2023, 33, 1140. 37594753 10.1089/thy.2022.0636

[13] J. A. Waung , J. H. Bassett , G. R. Williams , Trends Endocrinol. Metab. 2012, 23, 155. 22169753 10.1016/j.tem.2011.11.002

[14] H. Y. Kim , S. Mohan , Bone Res. 2013, 1, 146. 26273499 10.4248/BR201302004 PMC4472099

[15] I. Ross , D. B. Omengan , G. N. Huang , A. Y. Payumo , J. Endocrinol. 2022, 252, R71. 34935637 10.1530/JOE-21-0335 PMC8776588

[16] M. Puthenedam , F. Wu , A. Shetye , A. Michaels , K. J. Rhee , J. H. Kwon , Inflamm. Bowel Dis. 2011, 17, 260. 20812334 10.1002/ibd.21443 PMC2998582

[17] S. Wang , L. Fu , B. Wang , Y. Cai , J. Jiang , Y. B. Shi , BMC Genomics 2024, 25, 1260. 39736516 10.1186/s12864-024-11175-4 PMC11686881

[18] R. Liu , W. Fan , J. Hu , K. Xu , Z. Huang , Y. Liu , C. Sun , Sci. Rep. 2025, 15, 4121. 39901040 10.1038/s41598-025-88412-7 PMC11791035

[19] S. Chen , X. Luo , W. Wang , X. H. Chen , N. Ma , X. Y. Zhu , T. Zhou , Q. J. Gao , D. W. Zhao , Sci. Rep. 2024, 14, 32060. 39738470 10.1038/s41598-024-83719-3 PMC11685444

[20] A. J. Rosko , A. C. Birkeland , E. Bellile , K. J. Kovatch , A. L. Miller , C. C. Jaffe , A. G. Shuman , S. B. Chinn , C. L. Stucken , K. M. Malloy , J. S. Moyer , K. A. Casper , M. E. P. Prince , C. R. Bradford , G. T. Wolf , D. B. Chepeha , M. E. Spector , Ann. Surg. Oncol. 2018, 25, 1288. 29264671 10.1245/s10434-017-6278-4 PMC6002868

[21] T. R. Lamichhane , S. Paudel , B. K. Yadav , H. P. Lamichhane , J. Biol. Phys. 2019, 45, 107. 30810960 10.1007/s10867-018-9518-3 PMC6408566

[22] A. S. Pörings , T. Lowin , B. Dufner , J. Grifka , R. H. Straub , Sci. Rep. 2019, 9, 13235. 31519956 10.1038/s41598-019-49743-4 PMC6744488

[23] J. Yin , X. Xu , Y. Guo , C. Sun , Y. Yang , H. Liu , P. Yu , T. Wu , X. Song , Cell Death Discov. 2024, 10, 424. 39358326 10.1038/s41420-024-02181-2 PMC11447141

[24] K. M. Citrin , B. Chaube , C. Fernández‐Hernando , Y. Suárez , Trends Endocrinol. Metab. 2024, 12, S1043. 10.1016/j.tem.2024.11.004 PMC12159263 39672762

[25] H. C. Van Beeren , W. M. Jong , E. Kaptein , T. J. Visser , O. Bakker , W. M. Wiersinga , Endocrinology 2003, 144, 552. 12538616 10.1210/en.2002-220604

[26] Y. D. Chu , C. T. Yeh , Cells 2020, 9, 1730.32698392

[27] S. Y. Cheng , Thyroid 2020, 30, 8.31822204

[28] M. Brissova , K. Aamodt , P. Brahmachary , N. Prasad , J. Y. Hong , C. Dai , M. Mellati , A. Shostak , G. Poffenberger , R. Aramandla , S. E. Levy , A. C. Powers , Cell Metab. 2014, 19, 498. 24561261 10.1016/j.cmet.2014.02.001 PMC4012856

[29] Y. Fu , Y. Zhou , K. Wang , Z. Li , W. Kong , Circ. Res. 2024, 134, 931. 38547250 10.1161/CIRCRESAHA.123.324055

[30] D. Wang , J. Jiang , M. Wang , K. Li , H. Liang , N. Wang , W. Liu , M. Wang , S. Zhou , M. Zhang , Y. Xiao , X. Shen , Z. Li , W. Wu , X. Lin , X. Xiang , Q. Xie , W. Liu , X. Zhou , Q. Tang , W. Zhou , L. Yang , C. M. Chuong , M. Lei , M. P. H. R. A. G. Metabolism , Research 2024, 7, 0433. 39091635 10.34133/research.0433 PMC11292124

[31] R. J. Owens , F. E. Baralle , EMBO J. 1986, 5, 2825. 3024962 10.1002/j.1460-2075.1986.tb04575.x PMC1167230

[32] J. Calaycay , H. Pande , T. Lee , L. Borsi , A. Siri , J. E. Shively , L. Zardi , J. Biol. Chem. 1985, 260, 12136. 3900070

[33] A. Garcia‐Pardo , A. Rostagno , B. Frangione , Biochem. J. 1987, 241, 923. 3593230 10.1042/bj2410923 PMC1147649

[34] A. Rostagno , M. J. Williams , M. Baron , I. D. Campbell , L. I. Gold , J. Biol. Chem. 1994, 269, 31938. 7989369

[35] M. Barbariga , F. Vallone , E. Mosca , F. Bignami , C. Magagnotti , P. Fonteyne , F. Chiappori , L. Milanesi , P. Rama , A. Andolfo , G. Ferrari , Sci. Rep. 2019, 9, 14272. 31582785 10.1038/s41598-019-50718-8 PMC6776511

[36] P. Konieczny , Y. Xing , I. Sidhu , I. Subudhi , K. P. Mansfield , B. Hsieh , D. E. Biancur , S. B. Larsen , M. Cammer , D. Li , N. X. Landén , C. Loomis , A. Heguy , A. N. Tikhonova , A. Tsirigos , S. Naik , Science 2022, 377, abg9302. 10.1126/science.abg9302 PMC9753231 35709248

[37] M. Lei , H. I. Harn , Q. Li , J. Jiang , W. Wu , W. Zhou , T. X. Jiang , M. Wang , J. Zhang , Y. C. Lai , W. T. Juan , R. B. Widelitz , L. Yang , Z. Z. Gu , C. M. Chuong , Proc. Natl. Acad. Sci. USA 2023, 120, 2221982120. 10.1073/pnas.2221982120 PMC10483620 37643215

[38] M. Lei , J. Jiang , M. Wang , W. Wu , J. Zhang , W. Liu , W. Zhou , Y. C. Lai , T. X. Jiang , R. B. Widelitz , H. I. Harn , L. Yang , C. M. Chuong , NPJ Regen. Med. 2023, 8, 65. 37996466 10.1038/s41536-023-00340-0 PMC10667216

[39] S. Zhou , Z. Li , X. Li , Y. Ye , M. Wang , J. Jiang , L. Tao , Y. Wang , C. T. Tung , Y. Chung , E. Kim , X. Shen , X. Xu , X. Xiang , Q. Xie , J. Zhang , W. Wu , X. Lin , C. M. Chuong , M. Lei , J. Adv. Res. 2024, 70, 339. 38718895 10.1016/j.jare.2024.05.006 PMC11976415

[40] M. Wang , X. Zhou , S. Zhou , M. Wang , J. Jiang , W. Wu , T. Liu , W. Xu , J. Zhang , D. Liu , Y. Zou , W. Qiu , M. Zhang , W. Liu , Z. Li , D. Wang , T. Li , J. Li , W. Liu , L. Yang , M. Lei , Theranostics 2023, 13, 2930. 37284452 10.7150/thno.83217 PMC10240816

[41] M. Lei , L. J. Schumacher , Y. C. Lai , W. T. Juan , C. Y. Yeh , P. Wu , T. X. Jiang , R. E. Baker , R. B. Widelitz , L. Yang , C. M. Chuong , Proc Natl Acad Sci USA 2017, 114, E7101. 28798065 10.1073/pnas.1700475114 PMC5576784

[42] C. Yi , W. Wu , D. Zheng , G. Peng , H. Huang , Z. Shen , X. Teng , Cell Death Dis. 2020, 11, 533. 32665543 10.1038/s41419-020-02737-x PMC7360547

[43] B. Cao , Q. Xu , Y. Shi , R. Zhao , H. Li , J. Zheng , F. Liu , Y. Wan , B. Wei , Signal Transduct. Target. Ther. 2024, 9, 155. 38851750 10.1038/s41392-024-01845-w PMC11162504

[44] D. Jiang , H. G. Machens , Y. Rinkevich , Signal Transduct. Target. Ther. 2024, 9, 177. 39043659 10.1038/s41392-024-01880-7 PMC11266685

[45] A. Laurino , E. Landucci , F. Resta , G. De Siena , D. E. Pellegrini‐Giampietro , A. Masi , G. Mannaioni , L. Raimondi , Thyroid 2018, 28, 1387. 30129879 10.1089/thy.2017.0506

[46] C. Lin , N. Li , H. Chang , Y. Shen , Z. Li , W. Wei , H. Chen , H. Lu , J. Ji , N. Liu , Cell Death Dis. 2020, 11, 671. 32826870 10.1038/s41419-020-02836-9 PMC7442821

[47] J. D. Gothié , P. Vancamp , B. Demeneix , S. Remaud , Acta Physiol. 2020, 228, 13316. 10.1111/apha.13316 PMC9286394 31121082

[48] L. Sabatino , D. Lapi , C. Del Seppia , Biomolecules 2024, 14, 198. 38397435 10.3390/biom14020198 PMC10886502

[49] M. F. Yam , Y. C. Loh , C. S. Tan , S. Khadijah Adam , N. Abdul Manan , R. Basir , Int. J. Mol. Sci. 2018, 19, 2164. 30042373 10.3390/ijms19082164 PMC6121522

[50] D. Roosterman , T. Goerge , S. W. Schneider , N. W. Bunnett , M. Steinhoff , Physiol. Rev. 2006, 86, 1309. 17015491 10.1152/physrev.00026.2005

[51] L. Michalik , W. Wahli , J. Clin. Invest. 2006, 116, 598. 16511592 10.1172/JCI27958 PMC1386118

[52] R. Jin , L. Luo , J. Zheng , Life 2022, 12, 725. 35629392 10.3390/life12050725 PMC9144330

[53] C. Contreras‐Jurado , L. García‐Serrano , M. Gómez‐Ferrería , C. Costa , J. M. Paramio , A. Aranda , J. Biol. Chem. 2011, 286, 24079. 21566120 10.1074/jbc.M111.218487 PMC3129189

[54] B. Paulose , S. Chhikara , J. Coomey , H. I. Jung , O. Vatamaniuk , O. P. Dhankher , Plant Cell 2013, 25, 4580. 24214398 10.1105/tpc.113.111815 PMC3875737

[55] T. Ito , T. Kitaiwa , K. Nishizono , M. Umahashi , S. Miyaji , S. I. Agake , K. Kuwahara , T. Yokoyama , S. Fushinobu , A. Maruyama‐Nakashita , R. Sugiyama , M. Sato , J. Inaba , M. Y. Hirai , N. Ohkama‐Ohtsu , Plant J. 2022, 111, 1626. 35932489 10.1111/tpj.15912 PMC9804317

[56] W. Liu , J. Jiang , Z. Li , Y. Xiao , S. Zhou , D. Wang , Y. Zou , T. Liu , K. Li , H. Liang , N. Wang , X. Xiang , Q. Xie , R. Zhan , J. Zhang , X. Zhou , L. Yang , C. M. Chuong , M. Lei , Theranostics 2024, 14, 3339. 38855186 10.7150/thno.93764 PMC11155411

[57] Y. Ma , S. Shen , Y. Yan , S. Zhang , S. Liu , Z. Tang , J. Yu , M. Ma , Z. Niu , Z. Li , Y. Wu , L. Zhao , Z. Lu , C. Wei , W. J. Zhang , Y. Xue , Q. Zhai , Y. Li , C. Hu , J. Jiang , Y. Li , H. Ying , Diabetes 2023, 72, 562. 36724137 10.2337/db22-0656

[58] S. Su , Y. Zhang , D. Wu , C. Wang , J. Hu , Y. Wei , X. Peng , Burns Trauma 2024, 12, tkae007. 38756185 10.1093/burnst/tkae007 PMC11097601

[59] T. I. Mitchell , C. E. Brinckerhoff , Amyloid 1995, 2, 83.

[60] M. Fasshauer , J. Klein , S. Kralisch , M. Klier , U. Lossner , M. Bluher , R. Paschke , J. Endocrinol. 2004, 183, 561. 15072573 10.1677/joe.0.1810129

[61] J. Lee , W. H. van der Valk , S. A. Serdy , C. Deakin , J. Kim , A. P. Le , K. R. Koehler , Nat. Protoc. 2022, 17, 1266. 35322210 10.1038/s41596-022-00681-y PMC10461778

