# Supplementary material for: Spatiotemporal Adaptations‐Driven Dynamic Thra Activation Simulates a Skin Wound Healing Response
Source: Adv Sci (Weinh). 2025 Jun 25;12(34):e06651. doi: 10.1002/advs.202506651 (PMC12442676; doi:10.1002/advs.202506651)
Supplement: Supplementary file 1 — Supporting Information [file ADVS-12-e06651-s001.docx]

**Supplementary materials**


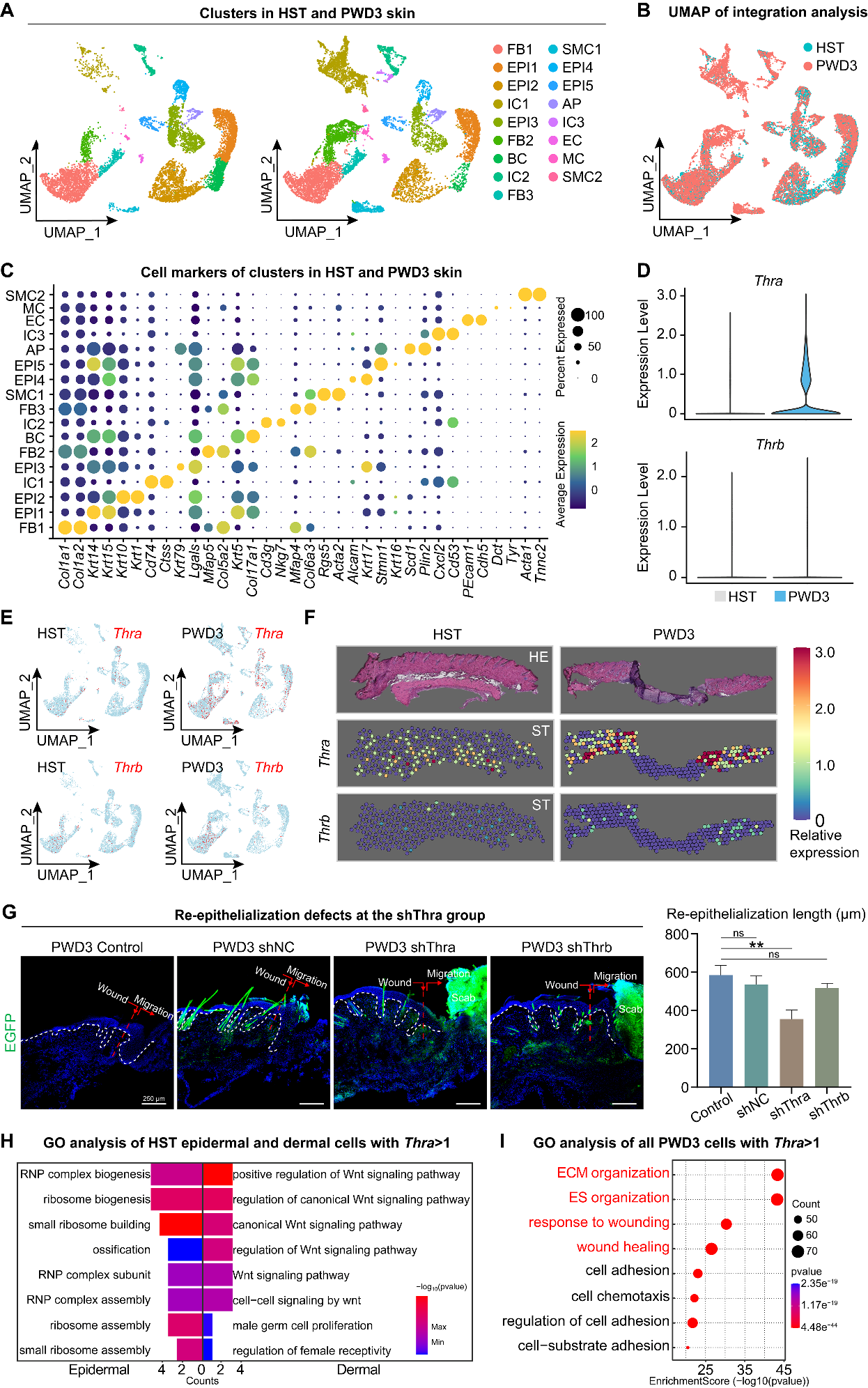


**Figure S1. Spatiotemporal expression of THRA upregulated on post-wound day 3.**

A. UMAPs of different clusters in HST and PWD3 skin.

B. UMAP of cell integration in HST and PWD3 skin.

C. DotPlot of the marker genes of different clusters.

D. Vlnplots of the expression of Thra and Thrb in HST and PWD3 skin.

E. FeaturePlots of the expression of Thra and Thrb in HST and PWD3 skin.

F. Spatial transcriptomics data of the expression of Thra and Thrb in HST and PWD3 skin.

G. Immunofluorescence images of EGFP expression in the control, shNC, shThrb, and shThra groups, with statistical analysis of re-epithelialization length. (Scale bars, 250 μm; N = 5, **p < 0.01, ns: no significance)

H. GO analysis of pathways enriched in Thra-positive cells in HST epidermal and dermal cells.

I. GO analysis of pathways enriched in Thra-positive cells among all cells in PWD3.\


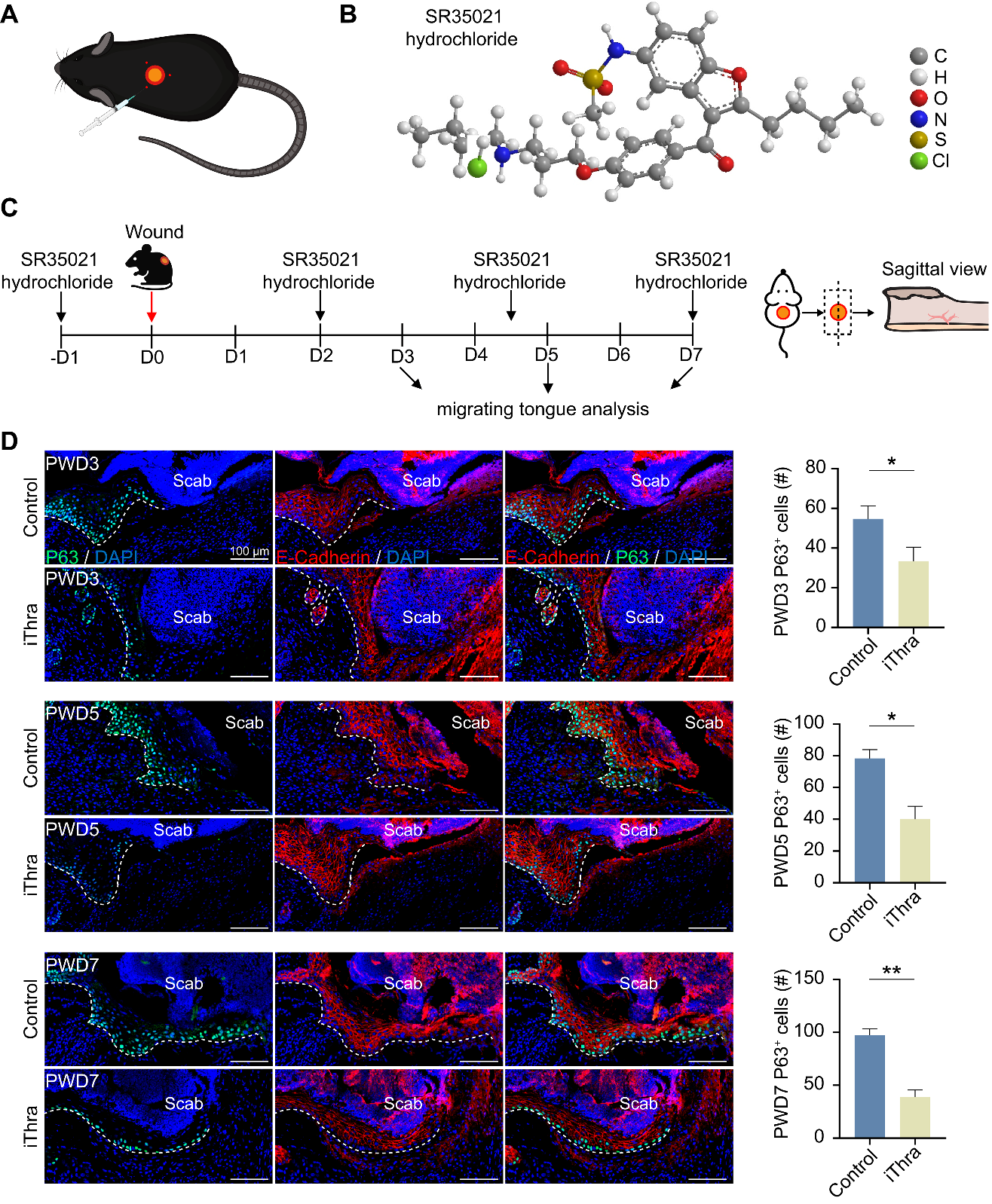


**Figure S2. Inhibition of Thra delayed epithelialization and dermal collagen deposition.**

A. Schematic illustration of the injection methods.

B. Molecular structure of SR35021.

C. Schematic illustration of experimental design for SR35021 injection.

D. Immunofluorescence images of E-Cadherin/P63 expression in the control and Thra inhibition groups on PWD3, PWD5 and PWD7, with statistical analysis of the average number of P63^+^ cells. (Scale bars, 100 μm; N = 5, **p* < 0.05)


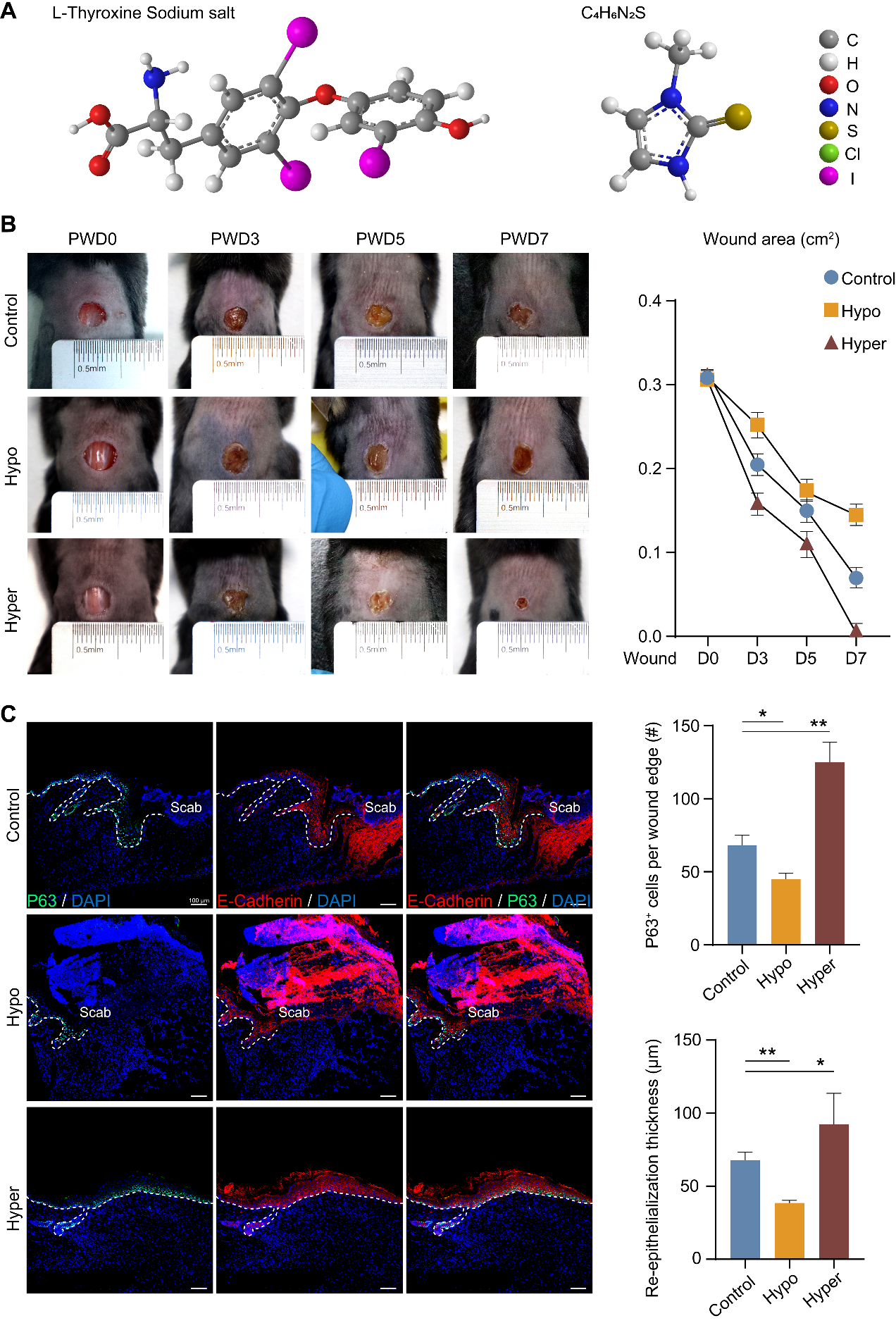


**Figure S3.** **High expression level of TH promoted wound repair.**

A. Molecular structure of L-Thyroxine sodium salt and methimazole.

B. Phase-contrast microscope images of wound healing process after thyroid dysfunction treatments, with statistical analysis of the average wound size. (Scale bars, 0.5 mm. N = 5)

C. Immunofluorescence images of E-Cadherin/P63 expression in the control, hypothyroidism and hyperthyroidism groups, with statistical analysis of the average numbers of P63^+^ cells. (Scale bars, 100 μm; N = 5, ***p* < 0.01, **p* < 0.05, ns: no significance)


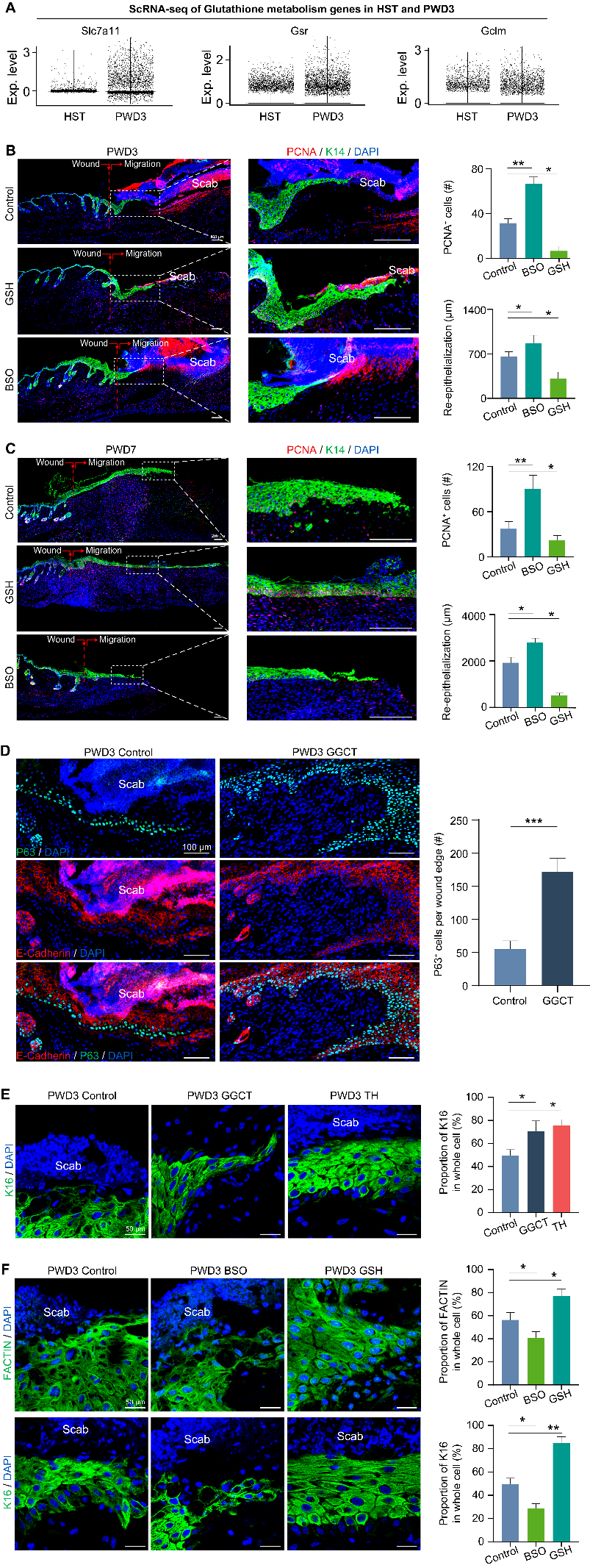


**Figure S4.** **Thra-regulated glutathione metabolism in the epidermis.**

A. Vlnplots of the gene expression of Slc7a11, Gsr, and Gclm in HST and PWD3 skin.

B. Immunofluorescence images of PCNA/K14 expression in the control, GSH and BSO groups, with statistical analysis of the average number of PCNA^+^ cells and the average re-epithelialization length on PWD3. (Scale bars, 200 μm; N = 5, ***p* < 0.01, **p* < 0.05)

C. Immunofluorescence images of PCNA/K14 expresion in the control, GSH and BSO groups, with statistical analysis of the average number of PCNA^+^ cells and the average re-epithelialization length on PWD7. (Scale bars, 200 μm; N = 5, ***p* < 0.01, **p* < 0.05)

D. Immunofluorescence images of E-Cadherin/P63 expression in the control and GGCT groups, with statistical analysis of the average number of P63^+^ cells on PWD3. (Scale bars, 100 μm; N = 5, ****p* < 0.001)

E. Immunofluorescence images of K16 expression in the control, GGCT and TH groups, with statistical analysis of the average proportion of K16 in total cells. (Scale bars, 50 μm; N = 5, **p* < 0.05)

F. Immunofluorescence images of FACTIN and K16 expressions in the control, GSH and BSO groups, with statistical analysis of the average proportion of FACTIN and K16 in total cells. (Scale bars, 50 μm; N = 5, ***p* < 0.01, **p* < 0.05)


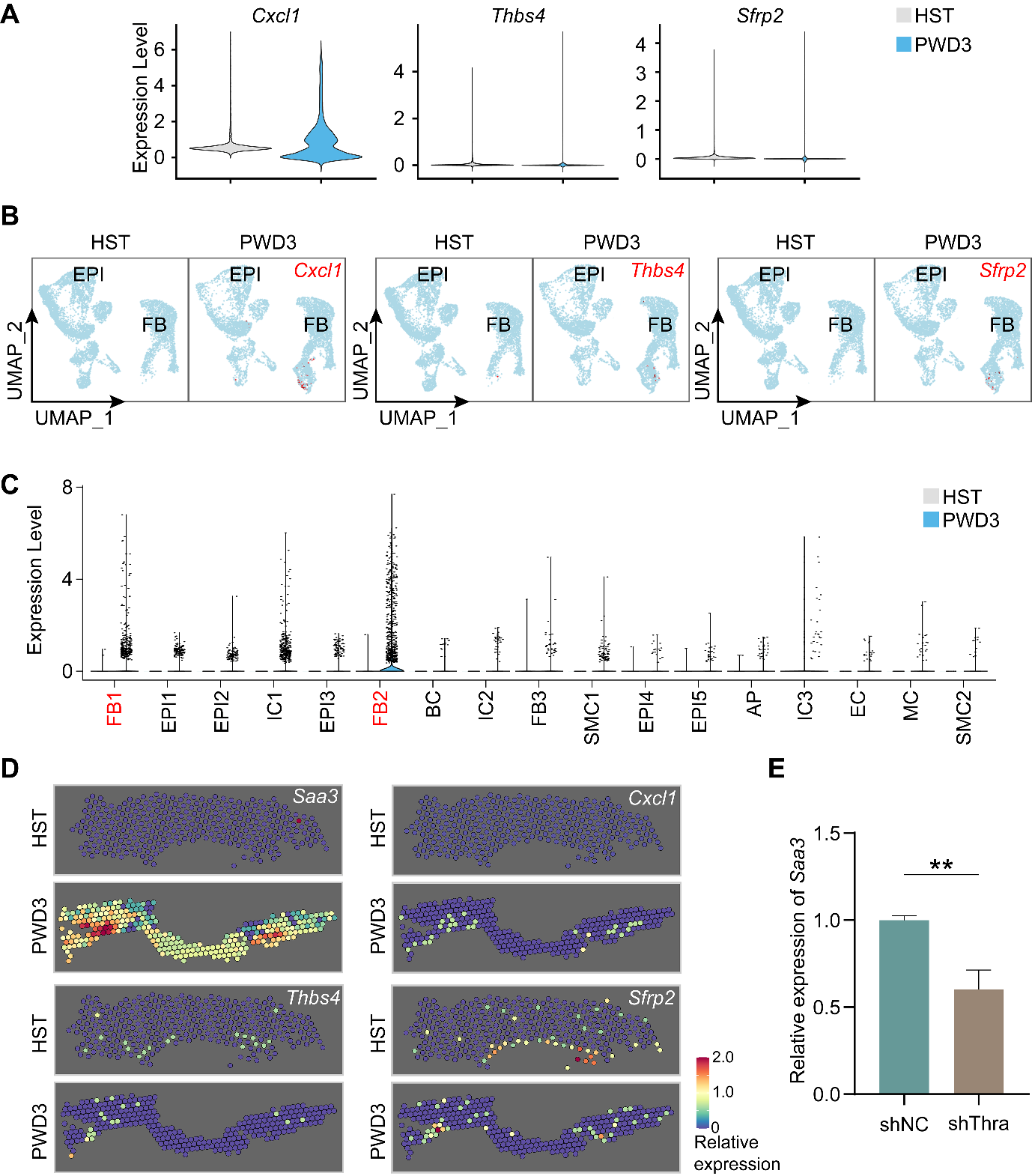


**Figure S5.** **Thra-activated SAA3 upregulated at the dermal wound edge.**

A. Vlnplots of the gene expression of Cxcl1, Thbs4, and Sfrp2 in HST and PWD3 skin.

B. FeaturePlots of the expression of Cxcl1, Thbs4, and Sfrp2 in HST and PWD3 skin within FB and EPI clusters.

C. VlnPlot of the expression of Saa3.

D Spatial transcriptomics data of the expression of Saa3, Cxcl1, Thbs4, and Sfrp2 in HST and PWD3 skin.

E. qRT-PCR analysis of Saa3 expression levels after Thra knockdown. (N = 3, **p < 0.01)


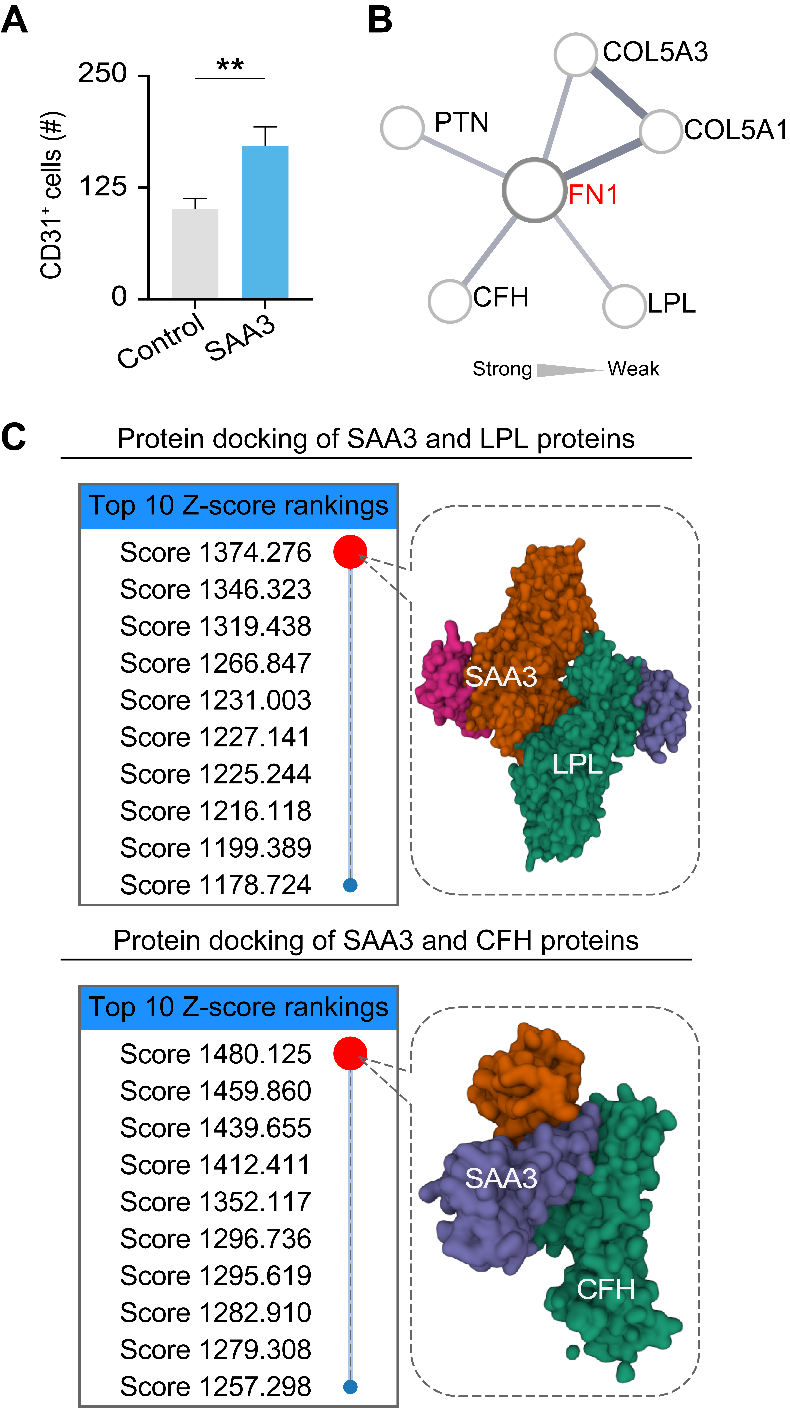


**Figure S6. SAA3 favored dermal Fn1 protein functions.**

A. Statistical analysis of CD31^+^ cells. (N= 5, ***p* < 0.01)

B. Protein network analysis of the six SAA3-related proteins.

C. Protein docking of the binding status of SAA3-LPL and SAA3-CFH.


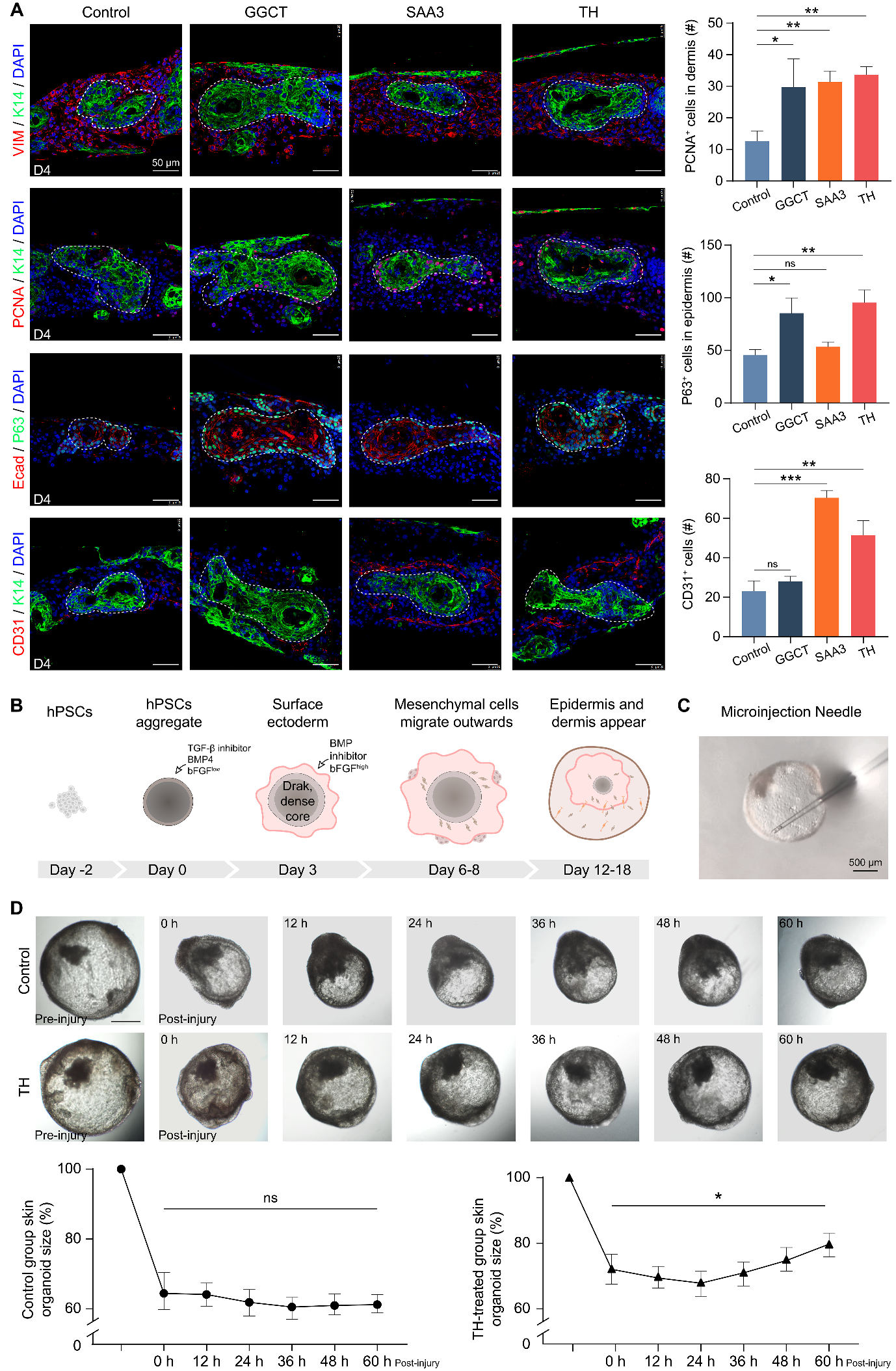


**Figure S7. GGCT, SAA3, and TH regulatory networks demonstrated in skin organoid models.**

**A.** Immunofluorescence images of VIM/K14, PCNA/K14, Ecad/P63, and CD31/K14 expressions on the fourth day of cultured skin organoids, with statistical analysis of the average number of PCNA^+^ cells in dermis, the average number of P63^+^ cells in epidermis and the average number of CD31^+^ cells. (Scale bars, 50 μm; N = 3, ***p* < 0.01, **p* < 0.05, ns: no significance)

B. Schematic illustration of the experimental design for human skin organoids.

C. Phase-contrast microscope images of microinjection needle. (Scale bars, 500 μm)

D. Phase-contrast microscope images of skin organoid size after TH treatment, with statistical analysis of the average skin organoid size. (Scale bars, 500 μm; N = 3, *p < 0.05, ns: no significance)


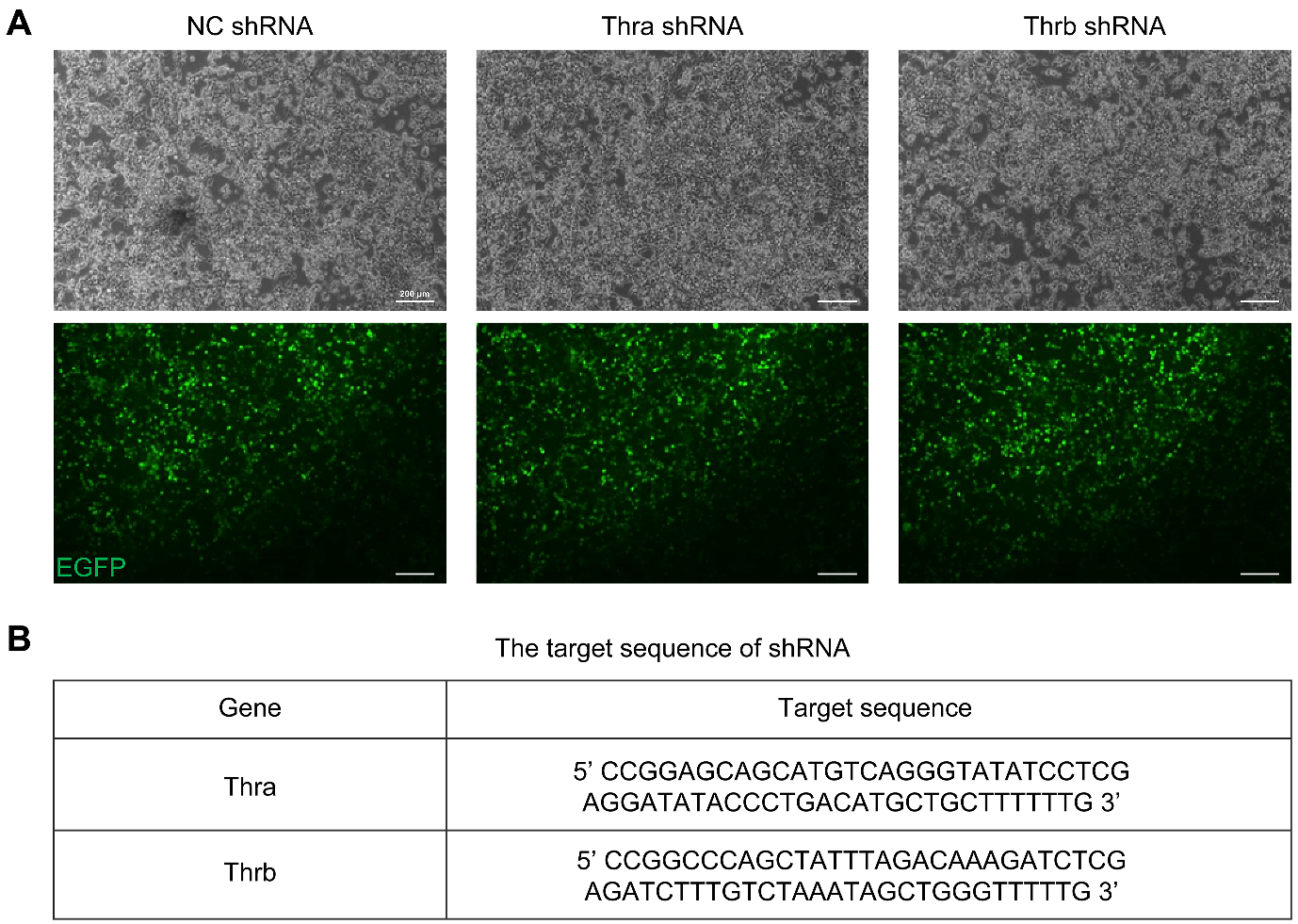


**Figure S8.** **Lentiviral shRNA knockdown.**

A. Transfection efficiency assessed via EGFP expression. (Scale bars, 200 μm)

B. The target sequence of shRNA.
